# A Bayesian adaptive design for clinical trials in rare diseases

**DOI:** 10.1016/j.csda.2016.09.006

**Published:** 2016-09-28

**Authors:** S. Faye Williamson, Peter Jacko, Sofía S. Villar, Thomas Jaki

**Affiliations:** a Department of Mathematics and Statistics, Lancaster University, UK; b Department of Management Science, Lancaster University, UK; c MRC Biostatistics Unit, Cambridge, UK

**Keywords:** Clinical trials, Rare diseases, Bayesian adaptive designs, Sequential allocation, Bandit models, Dynamic programming

## Abstract

Development of treatments for rare diseases is challenging due to the limited number of patients available for participation. Learning about treatment effectiveness with a view to treat patients in the larger outside population, as in the traditional fixed randomised design, may not be a plausible goal. An alternative goal is to treat the patients within the trial as effectively as possible. Using the framework of finite-horizon Markov decision processes and dynamic programming (DP), a novel randomised response-adaptive design is proposed which maximises the total number of patient successes in the trial and penalises if a minimum number of patients are not recruited to each treatment arm. Several performance measures of the proposed design are evaluated and compared to alternative designs through extensive simulation studies using a recently published trial as motivation. For simplicity, a two-armed trial with binary endpoints and immediate responses is considered. Simulation results for the proposed design show that: (i) the percentage of patients allocated to the superior arm is much higher than in the traditional fixed randomised design; (ii) relative to the optimal DP design, the power is largely improved upon and (iii) it exhibits only a very small bias and mean squared error of the treatment effect estimator. Furthermore, this design is fully randomised which is an advantage from a practical point of view because it protects the trial against various sources of bias. As such, the proposed design addresses some of the key issues that have been suggested as preventing so-called bandit models from being implemented in clinical practice.

## 1 Introduction

Before any new medical treatment is made available to the public, clinical trials must be undertaken to ensure that the treatment is safe and efficacious. Development of treatments for rare diseases is particularly challenging due to the limited number of patients available for experimentation.

The current gold standard design is the randomised controlled trial, in which patients are randomised to either the experimental or control treatment in a pre-fixed proportion. Its main goal is to learn about treatment effectiveness with a view to prioritising future patients outside of the trial. Although this design can detect a significant treatment difference with a high probability, i.e. it maximises the statistical power, which is of benefit to future patients, it lacks the flexibility to incorporate other desirable criteria, such as the trial participant’s well-being. As such, a large number of patients within the trial receive the inferior treatment. This is particularly concerning for rare disease trials in which a substantial proportion of all patients with the disease may be included in the trial. Moreover, there will be fewer patients available outside of the trial to benefit from the learning. Therefore, in this case, the priority should be on treating those patients within the trial as effectively as possible.

This motivates the use of response-adaptive designs for clinical trials involving rare diseases in which the accruing data on patient responses are used to skew the allocation towards the superior treatments, thus reducing patient exposure to inferior treatments. Although it does not fully eliminate the ethical problem of randomising patients to the inferior treatment, it certainly mitigates it by reducing the probability of allocation to the inferior treatment, if it exists.

Berry and Eick (1995) compare the performance of the traditional design, in which half of the participants receive treatment *A* and the other half receive treatment *B*, to four response-adaptive designs. They conclude that if the condition being treated is rare, then response-adaptive methods can perform substantially better and might be a more suitable alternative.

Despite the long history in clinical trials methodology, very few response-adaptive designs have actually occurred in practice and applications thus far have been disappointing (Rosenberger, 1999). This is largely attributable to the extracorporeal membrane oxygenation (ECMO) trial by Bartlett et al. (1985) which employed the randomised play-the-winner rule, a response-adaptive design described briefly in Section 2.

The problem of designing a clinical trial which aims to identify the superior treatment (exploration or learning) whilst treating the trial participants as effectively as possible (exploitation or earning) is a natural application area for bandit models, a type of response-adaptive design. Bandit models seek to balance the exploration versus exploitation trade-off in order to obtain an optimal allocation policy which maximises the expected number of patient successes over a finite number of patients. As such, they present an appealing alternative to the traditional approach used in clinical trials. Across the bandit literature, the use of bandit models to optimally design a clinical trial is often referred to as the primary motivation for their study (Gittins, 1979). However, to the best of our knowledge, they have never been implemented in real clinical practice for reasons including lack of randomisation and biased treatment effect estimates. Moreover, in contrast to the traditional approach taken in clinical trials, bandit models exhibit very low power since it is not possible to maximise both power and patient successes simultaneously. For a discussion of the benefits and challenges of bandit models in clinical trial practice, see Villar et al. (2015a).

In this paper, we propose a novel bandit-based design which provides a very appealing compromise between these two conflicting objectives and addresses some of the key issues that have prevented bandit models from being implemented in clinical trial practice. We modify the optimal design, which aims to maximise the expected number of patient successes, in such a way that we overcome its limitations without having a significant negative impact on the patient benefit.

The modifications involve incorporating randomisation into a currently deterministic design, which was considered by Cheng and Berry (2007), and adding a constraint which forces a minimum number of patients on each treatment. These are described in Sections 2.2 and 2.3, respectively, building on the standard dynamic programming approach presented in Section 2.1. In Section 4, we compare our design to alternative designs via extensive simulations in several scenarios in the context of a recently published Phase II clinical trial of isotonic fluid resuscitation in children with severe malnutrition and hypovolaemia (Akech et al., 2010). We evaluate each design’s performance according to the measures set out in Section 3. We summarise the main conclusions in Section 5 and highlight areas for future research.

## 2 Methods

In this section, we introduce different methods for allocating patients to treatments in a clinical trial. For simplicity of exposition, we consider a two-armed clinical trial with a binary endpoint and a finite number of patients within the trial, *n*. Patients enter the trial sequentially over time, one-by-one, and each patient is allocated to either treatment *A* or *B* on arrival. We assume that *n* is fixed but that the sample sizes for treatment groups *A* and *B*, denoted by *N_A_* and *N_B_* respectively, are random, where *N_A_* + *N_B_* = *n*. Let *X* and *Y* denote the patient’s response (either a success or failure) from treatments *A* and *B* respectively, which we model as independent Bernoulli random variables. That is,

$$X∼\text{Bernoulli}\left(1,\theta_{A}\right)\;\text{and}\;Y∼\text{Bernoulli}\left(1,\theta_{B}\right),\;\text{for}\;0\leq \theta_{A},\theta_{B}\leq 1,$$

where *θ_A_* and *θ_B_* are the unknown success probabilities of treatments *A* and *B* respectively. Further, assume that each patient’s response from the allocated treatment becomes immediately available.

The *fixed randomised* design randomises patients to either treatment *A* or *B* with an equal, fixed probability, i.e. 50% in a two-armed trial. This will act as a reference to which each of the response-adaptive designs described below will be compared against.

One of the most well-known response-adaptive designs is the *randomised play-the-winner* (RPW) rule, a type of urn model, proposed by Wei and Durham (1978). This design is very intuitive and applies specifically to clinical trials comparing two treatments with binary responses. Initially, an urn contains *u* balls of type *A* and *u* balls of type *B*. When a patient is recruited, a ball is drawn randomly from the urn with replacement; if it is a type *A* ball, the patient receives treatment *A* and if it is a type *B* ball, the patient receives treatment *B*. After each patient’s outcome is observed, a decision about the urn composition is made depending on the observed result. Thus, a success on treatment *A*, or a failure on treatment *B*, generates an additional *β* type *A* balls and *α* type *B* balls in the urn. Similarly, a success on treatment *B*, or a failure on treatment *A*, will generate an additional *β* type *B* balls and *α* type *A* balls in the urn, where 0 ≤ *α* ≤ *β* are integers. In this way, the urn accumulates more balls representing the superior treatment, thus increasing the probability that a patient receives the current best treatment. Note that the RPW is essentially myopic (as are most response-adaptive designs) in the sense that it uses all of the past observations to treat the next patient as if this were the last patient in the trial.

### 2.1 Optimal design using dynamic programming (DP)

The RPW described above is not constructed based on any formal optimality criterion so we now turn our attention to an alternative approach which utilises dynamic programming. With this approach, prior information on the unknown parameters is used in conjunction with the incoming data (and the number of remaining patients in the trial) to determine the optimal treatment allocation for every patient of the trial.

Note that we use *t* to denote both time and the last patient treated in this model since they are analogous, that is, at time *t* we have treated *t* patients. The trial time is therefore bounded by 0 ≤ *t* ≤ *n*.

Since the treatment effects take values between zero and one, it is sensible to assign the parameters independent Beta prior distributions

$$\theta_{A}∼\text{Beta}\left(s_{A,0},f_{A,0}\right)\;\text{and}\;\theta_{B}∼\text{Beta}\left(s_{B,0},f_{B,0}\right)\;\text{for}\;0\leq \theta_{A},\theta_{B}\leq 1.$$

Since this is a conjugate prior with respect to the Bernoulli likelihood function, the posterior distribution follows another Beta distribution with parameters summarising the relevant information from the trial to date (that is, the combination of the initial prior plus the accumulated data). At time *t* ≥ 1, after observing *s_A,t_* (*f_A,t_*) successes (failures) on treatment *A*, and *s_B,t_* (*f_B,t_*) successes (failures) on treatment *B*, the posterior distribution is expressed by

$$\theta_{A}|s_{A,t},f_{A,t}∼\text{Beta}\left(s_{A,0}+s_{A,t},\;f_{A,0}+f_{A,t}\right)\;\text{and}\;\theta_{B}|s_{B,t},f_{B,t}∼\text{Beta}\left(s_{B,0}+s_{B,t},\;f_{B,0}+f_{B,t}\right),$$

where *s_A,t_* + *f_A,t_* + *S_B,t_* + *f_B,t_* = *t* for *t* ≥ 1. Therefore, it will only be necessary to update the parameters of these distributions as the trial progresses. For simplicity, let the prior information and data combined be denoted as

(1) $${\tilde{s}}_{A,t}=s_{A,0}+s_{A,t},\;{\tilde{f}}_{A,t}=f_{A,0}+f_{A,t},\;{\tilde{s}}_{B,t}=s_{B,0}+s_{B,t}\;\text{and}\;{\tilde{f}}_{B,t}=f_{B,0}+f_{B,t}.$$

Therefore, $\frac{{\tilde{s}}_{j,t}}{{\tilde{s}}_{j,t}+{\tilde{f}}_{j,t}}$ is the posterior probability (i.e. the *current belief*) of success for treatment *j* given the prior information and data up to patient *t*.

Let *δ_j,t_*, for *t* = 0, … , *n* − 1, be the binary indicator variable representing whether patient *t* + 1 is allocated to treatment *j* ∈ {*A, B*}, where

(2) $$\delta_{j,t}=\{\begin{array}{ll} 1, & \text{if}\;\text{patient}\;t+1\;\text{is}\;\text{allocated}\;\text{to}\;\text{treatment}\;j,\\ 0, & \text{otherwise}. \end{array}$$

Using the jargon of dynamic programming, *δ_j,t_* is the reward for every successfully treated patient, and thus $\frac{{\tilde{s}}_{j,t}}{{\tilde{s}}_{j,t}+{\tilde{f}}_{j,t}}\cdot \delta_{j,t}$ is the expected (one-period) reward, where expectation is taken in the Bayesian sense, i.e. according to the current belief.

Let *Π* be the family of admissible designs (i.e. allocation policies) *π*, which are those such that $\sum_{j}\delta_{j,t}=1$ for all *t* since only one treatment is allocated per patient. Let *ℱ_t_* (*s_A_*, *f_A_*, *s_B_*, *f_B_*) be the value function representing the maximum expected total reward, i.e. the maximum Bayes-expected number of successes, in the rest of the trial after *t* patients have been treated when the combined information is (*s_A_*, *f_A_*, *s_B_*, *f_B_*),

$${ℱ}_{t}\left(s_{A},f_{A},s_{B},f_{B}\right):=\underset{\pi \in \prod }{\text{max}{𝔼}^{\pi }}\left[\sum_{u=t}^{n-1}\sum_{j\in \{A,B\}}\frac{{\tilde{s}}_{j,u}}{{\tilde{s}}_{j,u}+{\tilde{f}}_{j,u}}\cdot \delta_{j,u}|{\tilde{s}}_{A,t}=s_{A},{\tilde{f}}_{A,t}=f_{A},{\tilde{s}}_{B,t}=s_{B},{\tilde{f}}_{B,t}=f_{B}\right].$$

Note that this depends on the total number of patients *n* even though we do not state it explicitly to simplify the notation.

The ultimate optimisation problem is to find an optimal design which maximises the expected total reward, i.e. the Bayes-expected number of successes, over the set of all policies in the whole trial for a given prior at time *t* = 0, namely,

(3) $${ℱ}_{0}\left(s_{A,0},f_{A,0},s_{B,0},f_{B,0}\right).$$

The problem summarised in Eq. (3) is known as a *finite-horizon Bayesian Bernoulli two-armed bandit problem* which can be solved exactly using dynamic programming methods, giving rise to an optimal adaptive treatment allocation sequence. Specifically, one can implement a backward induction algorithm which starts with the last patient, patient *n*, and proceeds iteratively towards the first patient. Details of this algorithm can be found in Appendix A.1.

Suppose that *t* < *n*. If treatment *A* is allocated to the next patient, then the expected total reward, i.e. the Bayes-expected number of successes, for patients *t* + 1 to *n* under an optimal policy is

$${ℱ}_{t}^{A}\left(s_{A},f_{A},s_{B},f_{B}\right)=\frac{s_{A}}{s_{A}+f_{A}}\cdot \left[1+{ℱ}_{t+1}\left(s_{A}+1,f_{A},s_{B},f_{B}\right)\right]+\frac{f_{A}}{s_{A}+f_{A}}\cdot {ℱ}_{t+1}\left(s_{A},f_{A}+1,s_{B},f_{B}\right).$$

Alternatively, if treatment *B* is allocated to the next patient, then the expected total reward, i.e. the Bayes-expected number of successes, for patients *t* + 1 to *n* under an optimal policy is

$${ℱ}_{t}^{B}\left(s_{A},f_{A},s_{B},f_{B}\right)=\frac{s_{B}}{s_{B}+f_{B}}\cdot \left[1+{ℱ}_{t+1}\left(s_{A,}f_{A},s_{B}+1,f_{B}\right)\right]+\frac{f_{B}}{s_{B}+f_{B}}.{ℱ}_{t+1}\left(s_{A},f_{A},s_{B},f_{B}+1\right).$$

Therefore, the value function satisfies the following recurrence known as the *principle of optimality*,

(4) $$\begin{array}{l} \begin{array}{l} {ℱ}_{t}\left(s_{A},f_{A},s_{B},f_{B}\right)=\text{max}\left\{{ℱ}_{t}^{A}\left(s_{A},f_{A},s_{B},f_{B}\right),\;{ℱ}_{t}^{B}\left(s_{A},f_{A},s_{B},f_{B}\right)\right\},\;\text{for}\;0\leq t\leq n-1,\\ {ℱ}_{n}\left(s_{A},f_{A},s_{B},f_{B}\right)=0,\;\text{otherwise}. \end{array} \end{array}$$

Unlike most response-adaptive designs, this is not a myopic allocation rule. Instead, all possible sequences of treatment allocations and responses are enumerated, and the sequence that maximises the expected number of patient successes over the finite planning horizon is selected (Hu and Rosenberger, 2006). As such, this approach is computationally intensive and suffers from the curse of dimensionality (Bellman, 1961). However, we provide an efficient algorithm for the optimal DP design, implemented in the statistical software R; the computational times are shown in Table A.2 of Appendix A.1.

The computational complexity of the dynamic programming methods to solve this problem is the main motivation behind the implementation of simpler index-based solutions which circumvent the aforementioned problem of dimensionality. One such solution, which we include as a comparator, is the *Whittle index* (WI) proposed by Whittle (1988). This can be applied when the horizon is finite, which is the case with a clinical trial since there are a finite number of patients in the trial. It is derived from a relaxation of problem (3), allowing the multi-armed problem to be decomposed into single-armed problems in which the states are augmented, adding the number of patients remaining to be treated as an additional state. Although the WI is a heuristic solution, it has been found to be near-optimal in several cases. See Villar et al. (2015a) for a detailed review of the WI as a potential patient allocation rule in a clinical trial.

It is shown in Villar et al. (2015a,b), and further illustrated by our results, that optimal designs which achieve the highest patient benefit suffer from very low power. Moreover, optimal designs are completely deterministic (Cheng and Berry, 2007) which means there is a risk of introducing various sources of bias into the trial, e.g. selection bias (Blackwell and Hodges, 1957). Both of these factors contribute to making the optimal design unsuitable to implement in clinical trial practice. Therefore, in the rest of this section we focus on modifications to the DP design which address these shortcomings, i.e. its determinism and low power, while improving over a fixed randomised design in terms of patient benefit measures, such as overall response.

### 2.2 Optimal design using randomised dynamic programming (RDP)

Randomisation is a critical component in the design of clinical trials, not least to minimise the bias and confounding of results in order to achieve the desired accuracy and reliability (Chow and Liu, 2014). Therefore, a natural first step is to modify the optimal design by forcing actions to be randomised; see Cheng and Berry (2007). This is achieved by assigning a probability to the allocation rule at each stage. In particular, we define the following actions so that each treatment has a probability of at least 1 − *p* of being allocated to each patient, where 0.5 ≤ *p* ≤ 1 for two-armed trials and will be referred to as the degree of randomisation. Note that *p* = 0.5 and *p* = 1 correspond to fixed, equal randomisation and the DP design, respectively.

(i) Action 1 (*a* = 1): The next patient receives treatment *A* with probability *p* and treatment *B* with probability 1 − *p*.
(ii) Action 2 (*a* = 2): The next patient receives treatment *B* with probability *p* and treatment *A* with probability 1 − *p*.

The associated expected total reward under this new action definition changes, along with the corresponding value function. Specifically, the expected total reward, i.e. the Bayes-expected number of successes, for patients *t* + 1 to *n* when *a* = 1 is now given by

$${ℱ}_{t}^{1}\left(s_{A},f_{A},s_{B},f_{B}\right)=p\cdot {ℱ}_{t}^{A}\left(s_{A},f_{A},s_{B},f_{B}\right)+\left(1-p\right)\cdot {ℱ}_{t}^{B}\left(s_{A},f_{A},s_{B},f_{B}\right),$$

and analogously when *a* = 2,

$${ℱ}_{t}^{2}\left(s_{A},f_{A},s_{B},f_{B}\right)=(1-p)\cdot {ℱ}_{t}^{A}\left(s_{A},f_{A},s_{B},f_{B}\right)+p\cdot {ℱ}_{t}^{B}\left(s_{A},f_{A},s_{B},f_{B}\right).$$

Thus, in contrast to that shown in (4), the value function satisfies

$$\begin{array}{l} {ℱ}_{t}\left(s_{A},f_{A},s_{B},f_{B}\right)=\text{max}\left\{{ℱ}_{t}^{1}\left(s_{A},f_{A},s_{B},f_{B}\right),{ℱ}_{t}^{2}\left(s_{A},f_{A},s_{B},f_{B}\right)\right\},\;\text{for}\;0\leq t\leq n-1,\\ {ℱ}_{n}\left(s_{A},f_{A},s_{B},f_{B}\right)=0,\;\text{otherwise}. \end{array}$$

We refer to this design as the *randomised dynamic programming* (RDP) design hereafter.

Preferably, we would like *p* to be as close to one as possible so that the action that allocates to the superior treatment with probability *p* is as effective as possible. However, this would entail that sometimes, by chance, the inferior treatment is sampled too few times or not at all. The possibility of this undesirable event occurring makes this design unsuitable to implement in practice as it results in low power and largely biased estimates.

### 2.3 Optimal design using constrained randomised dynamic programming (CRDP)

In order to circumvent having few or no observations on a treatment, we modify the optimal design further by adding a constraint to ensure that we always obtain at least *𝓁* observations from each treatment arm, where *𝓁* is a fixed predefined value and will be referred to as the degree of constraining. To do this, we add a penalty to the reward function for every combination of the states that give rise to less than *𝓁* observations on a treatment arm at the end of the trial.

We formulate this model as a Markov decision process with the following elements:

(i) Let $z_{t}=({\tilde{s}}_{A,t},{\tilde{f}}_{A,t},{\tilde{s}}_{B,t},{\tilde{f}}_{B,t},\tilde{n})$ be the vector of states representing all the information that is needed in order to choose an action for patient *t*, where ${\tilde{s}}_{A,t},{\tilde{f}}_{A,t},{\tilde{s}}_{B,t},{\tilde{f}}_{B,t}$ are as defined previously in (1), and $\tilde{n}=n-1$ is the number of patients in the trial remaining to be treated.
(ii) The action set, 𝓐; = {1, 2}, is composed of Action 1 (*a* = 1) and Action 2 (*a* = 2) as defined in Section 2.2.
(iii) The expected (one-period) reward under action *a* is given by ${ℛ}^{a}({\tilde{s}}_{A,t},{\tilde{f}}_{A,t},{\tilde{s}}_{B,t},\tilde{n}).$ If we are not at the end of the trial ($\tilde{n}\geq 1.$ then
  $${ℛ}^{a}({\tilde{s}}_{A,t},{\tilde{f}}_{A,t},{\tilde{s}}_{B,t},{\tilde{f}}_{B,t},\tilde{n}\geq 1)=\{\begin{array}{cc} p\;.\frac{{\tilde{s}}_{A,t}}{{\tilde{s}}_{A,t}+{\tilde{f}}_{A,t}}+(1-p)\;.\frac{{\tilde{s}}_{B,t}}{{\tilde{s}}_{B,t}+{\tilde{f}}_{B,t}}, & \text{if}\;a=1,\\ (1-p)\;.\frac{{\tilde{s}}_{A,t}}{{\tilde{s}}_{A,t}+{\tilde{f}}_{A,t}}+p\;.\frac{{\tilde{s}}_{B,t}}{{\tilde{s}}_{B,t}+{\tilde{f}}_{B,t}}, & \text{if}\;a=2. \end{array}$$
  Otherwise, if we are at the end of the trial with no more patients left to treat $\left(\tilde{n}=0\right)$, then
  $$ℛ({\tilde{s}}_{A,t},{\tilde{f}}_{A,t},{\tilde{s}}_{B,t},{\tilde{f}}_{B,t},\tilde{n}=0)=\{\begin{array}{cc} -n, & \text{if}\;s_{A,t}+f_{A,t}\;<\;l\;\text{or}\;s_{B,t}+f_{B,t}\;<l,\\ 0, & \text{otherwise}, \end{array}$$
  where –*n* is the penalty chosen because it is a large negative value which will cause the algorithm to avoid the undesirable states.
(iv) The non-zero transition probabilities, $\mathbb{P}\left({\text{z}}_{t+1}|{\text{z}}_{t},a\right),$ representing the evolution of the states from patient *t* to *t* + 1 under Action 1 and Action 2, respectively, are given as follows (where w.p. means “with probability”).
  When *a* = 1:
  $$z_{t+1}=\{\begin{array}{lll} \left({\tilde{s}}_{A,t}+1,{\tilde{f}}_{A,t},{\tilde{s}}_{B,t},{\tilde{f}}_{B,t},\tilde{n}-1\right) & \text{w}.\text{p}. & p.\frac{{\tilde{s}}_{A,t}}{{\tilde{s}}_{A,t}+{\tilde{f}}_{A,t}},\\ \left({\tilde{s}}_{A,t},{\tilde{f}}_{A,t}+1,{\tilde{s}}_{B,t},{\tilde{f}}_{B,t},\tilde{n}-1\right) & \text{w}.\text{p}. & p.\frac{{\tilde{f}}_{A,t}}{{\tilde{s}}_{A,t}+{\tilde{f}}_{A,t}},\\ \left({\tilde{s}}_{A,t},{\tilde{f}}_{A,t},{\tilde{s}}_{B,t}+1,{\tilde{f}}_{B,t},\tilde{n}-1\right) & \text{w}.p. & (1-p).\frac{{\tilde{s}}_{B,t}}{{\tilde{s}}_{B,t}+{\tilde{f}}_{B,t}},\\ \left({\tilde{s}}_{A,t},{\tilde{f}}_{A,t},{\tilde{s}}_{B,t},{\tilde{f}}_{B,t}+1,\tilde{n}-1\right) & \text{w}.\text{p}. & (1-p).\frac{{\tilde{f}}_{B,t}}{{\tilde{s}}_{B,t}+{\tilde{f}}_{B,t}}. \end{array}$$
  When *a* = 2:
  $$z_{t+1}=\{\begin{array}{lll} \left({\tilde{s}}_{A,t}+1,{\tilde{f}}_{A,t},{\tilde{s}}_{B,t},{\tilde{f}}_{B,t},\tilde{n}-1\right) & \text{w}.\text{p}. & (1-p).\frac{{\tilde{s}}_{A,t}}{{\tilde{s}}_{A,t}+{\tilde{f}}_{A,t}},\\ \left({\tilde{s}}_{A,t},{\tilde{f}}_{A,t}+1,{\tilde{s}}_{B,t},{\tilde{f}}_{B,t},\tilde{n}-1\right) & \text{w}.\text{p}. & (1-p).\frac{{\tilde{f}}_{A,t}}{{\tilde{s}}_{A,t}+{\tilde{f}}_{A,t}},\\ \left({\tilde{s}}_{A,t},{\tilde{f}}_{A,t},{\tilde{s}}_{B,t}+1,{\tilde{f}}_{B,t},\tilde{n}-1\right) & \text{w}.\text{p}. & p.\frac{{\tilde{s}}_{B,t}}{{\tilde{s}}_{B,t}+{\tilde{f}}_{B,t}},\\ \left({\tilde{s}}_{A,t},{\tilde{f}}_{A,t},{\tilde{s}}_{B,t},{\tilde{f}}_{B,t}+1,\tilde{n}-1\right) & \text{w}.\text{p}. & p.\frac{{\tilde{f}}_{B,t}}{{\tilde{s}}_{B,t}+{\tilde{f}}_{B,t}}. \end{array}$$

We refer to our proposed design as the *constrained randomised dynamic programming* (CRDP) design hereafter. For details of how this design was implemented in R, refer to the supplementary material which can be found online at http://dx.doi.org/10.1016/j.csda.2016.09.006.

## 3 Simulation set-up

We implement all of the above designs in several two-arm trial scenarios via simulations which will now be discussed, along with the performance measures that we use to compare and evaluate each design. The scenarios created are motivated by a recently published trial, as reported by Akech et al. (2010), which evaluated the effect of two different resuscitation treatments for children aged over six months with severe malnutrition and shock. The aim of the trial was to recruit 90 eligible patients, where 45 would be randomly assigned to group 0 (low dose hypotonic fluid: HSD/5D) and 45 to group 1 (Ringer’s Lactate: RL). The original trial allocated patients between the two arms with a fixed and equal randomisation probability of 0.5. The primary response outcomes were binary and available at eight and 24 h after randomisation (resolution of shock by 8/24 h). For this trial, 61 children were recruited, 26 received arm 0 and 29 received arm 1. At the end of the trial, the success rates observed in groups 0 and 1 at eight hours were 32% and 44%, respectively, and at 24 h were 22% and 44%, respectively. Although these differences were not statistically significant, the relatively quickly observed primary endpoint, the life-threatening nature of the disease, and the fact that patient recruitment is challenging, makes this trial an ideal motivating scenario for testing our proposed design.

Assuming that we begin the trial (at *t* = 0) in a state of equipoise, that is, a state of genuine uncertainty about which treatment is superior, we let *s*_*A*,0_ = *f*_*A*,0_ = *s*_*B,0*_ = *f*_*B,*0_ = 1, reducing this to a uniform prior.

We consider the following hypothesis

$$H_{0}:\theta_{A}=\theta_{B}\;\text{versus}\;H_{1}:\;\theta_{A}\neq \theta_{B},$$

which will be tested using Fisher’s exact test (Routledge, 2005) for comparing the success probabilities of two binomial distributions. Fisher’s exact test is probably the most common choice for binary outcomes and a small sample size. This test is a conditional test (conditioning on the marginals), which increases the discreteness and thus the conservatism of the test (Kateri, 2014). This means that the observed rejection rate is often far below the nominal significance level. Therefore, we set the nominal significance level to 0.1 throughout so that the observed type I error value will be closer to 0.05.

Alternatively, we could have followed a Bayesian inference procedure. However, in a clinical trial context a traditional hypothesis test is expected (due to both this being a common practice and because of regulatory requirements). Also, since all the simulations included in this paper use an uninformative prior, the impact of using a Bayesian estimator instead of the sample proportion for point estimation and decision making would be negligible.

In order to create a comprehensive picture of our proposed design, we run our simulations for a range of combinations of the success probability parameters *θ_A_* and *θ_B_*. Specifically, we consider *θ_A_* = 0.2 against *θ_B_* = (0.1, 0.2, … , 0.9), and similarly for *θ_A_* = 0.5 and 0.8. In the following, we focus on the scenario where *θ_A_* is fixed at 0.5 for all *θ_B_* ∈ (0.1, 0.9) since the patterns observed for the other cases are very similar.

Furthermore, we repeat the simulations for different total sample sizes. The results for *n* = 75 are reported throughout because this shows a good range of power values across all scenarios and clearly highlights the differences between each design, thus enabling us to make better comparisons. The results for *n* = 25, 50 and 100 are shown in Figs. A.10–A.12 of Appendix A.6.

We evaluate the performance of these designs by simulating 10,000 replications of each trial and taking the average values over these runs.

### 3.1 Performance measures

In addition to the operating characteristics, such as the power and type I error rate, we also consider the ethical performance of each design since this is one of the major advantages of response-adaptive designs over traditional fixed designs. Specifically, the criteria we focus on to assess the performance of each design are:

1. **Power**. The proportion of times we *correctly* reject *H*_0_ in the 10,000 trial replicas, i.e. the probability of making the correct decision at the end of the trial, so we want this to be high. This provides an informative measure of how well a test performs. This is calculated when *θ_A_* ≠ *θ_B_*.
2. **Type I error rate**. The proportion of times we *incorrectly* reject *H*_0_, i.e. the probability of making the incorrect decision at the end of the trial, so we want this to be low. This is calculated when *θ_A_* = *θ_B_*.
3. **Percentage of patients allocated to the superior treatment arm**. This measures the ethical performance of each design, which we wish to maximise.
4. **Average bias of the estimator**. This provides a measure of bias in the estimate of the treatment effect, where we define treatment effect as the treatment difference, $\hat{\Delta }={\hat{\theta }}_{A}-{\hat{\theta }}_{B}.$ The estimator of *θ_A_* and *θ_B_* is simply the sample proportion ${\hat{\theta }}_{A}=s_{A,n}/N_{A}\;\text{and}\;{\hat{\theta }}_{B}=s_{B,n}/N_{B},$ respectively. This is the observed proportion of successes in either treatment group by the end of the trial (at time *t* = *n*). The average bias of this estimator is defined to be the difference between the estimated success probability difference and the true success probability difference, that is,
  (5) $$\text{Bias}(\hat{\Delta })=𝔼(\hat{\Delta }-\Delta )=({\hat{\theta }}_{A}-{\hat{\theta }}_{B})-(\theta_{A}-\theta_{B}).$$
5. **Mean squared error (MSE) of the estimator**. The MSE provides a measure of the quality and variability of the estimator, $\hat{\Delta }$, and is defined by
  $$\text{MSE(}\hat{\Delta }\text{)}=𝔼\left[(\hat{\Delta }-\Delta {)}^{2}\right],$$
  which can be expressed in terms of the bias and variance of the estimator as,
  (6) $$\text{MSE}(\hat{\Delta })=\text{Bias}{\left(\hat{\Delta }\right)}^{2}+\text{Var}(\hat{\Delta }).$$

## 4 Simulation results and design comparison

We compare our proposed design to the alternative designs outlined in Section 2 based upon the performance measures highlighted in Section 3.1. We set *p* = 0.9 as the degree of randomisation and *𝓁* = 0.15*n* as the degree of constraining in our proposed CRDP design, which we believe yields robust design characteristics for many scenarios of interest and could be used as a quick rule of thumb. Alternatively, *𝓁* could be heuristically determined as the minimum sample size per arm required to attain a power of (1 − *γ*) in a fixed randomised design, where (1 − *γ*) ≤ (1 − *β*) and (1 − *β*) is the power level obtained by a fixed randomised trial of size *n*. In the following two paragraphs, we describe a more formal heuristic approach to determine *p* and *𝓁* when higher precision is needed to trade-off power and patient benefit.

We tried a range of values for *𝓁* ∈ (0.05*n*, 0.50*n*) (where 0.50*n* corresponds to fixed equal randomisation) and found that as *𝓁* increases, the power of the design increases hyperbolically, while the percentage of patients allocated to the superior treatment decreases linearly. This is illustrated in Fig. A.8 of Appendix A.2. We recommend choosing *𝓁* ∈ (0.10*n,* 0.15*n*) because for values of *𝓁* < 0.10*n*, the power is insufficient, and for values of *𝓁* > 0.15*n*, the very small gains in power do not outweigh the considerable reduction in the percentage of patients allocated to the superior treatment.

Similarly, we tried a range of values for *p* ∈ (0.5, 1) (where *p* = 0.5 and *p* = 1 correspond to fixed equal randomisation and the DP design, respectively) and found that there is only a slight decrease in power, but a very large increase in the percentage of patients allocated to the superior treatment as *p* increases from 0.5 to 0.9; see Tables A.3–A.6 in Appendix A.3. Therefore, we take *p* = 0.9 since this produces a good balance between the power and patient benefit across a wide range of scenarios and sample sizes.

### 4.1 Power and type I error

**Fig. 1:**
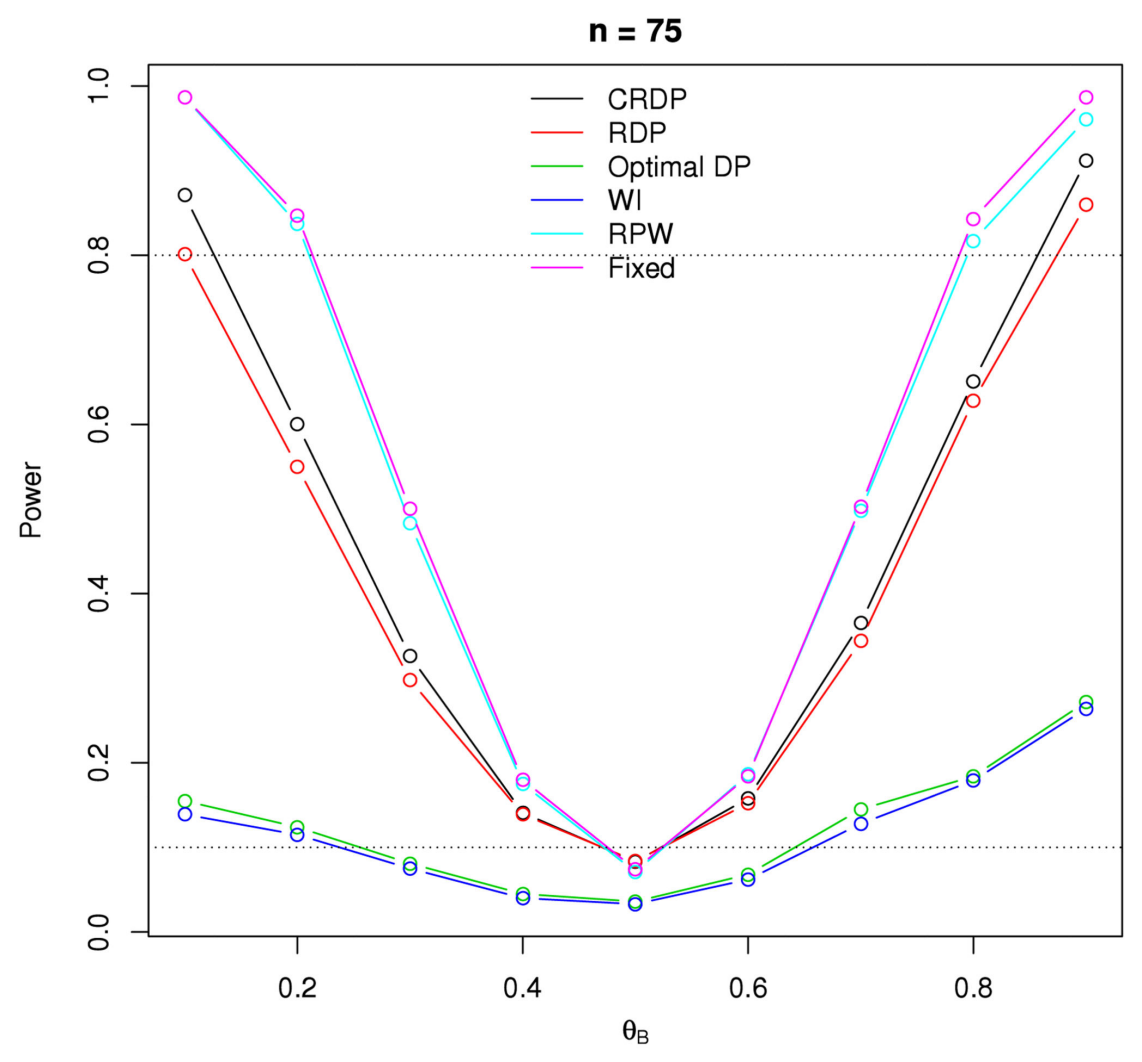
The changes in power and type I error for each design when *n* = 75, *θ_A_* = 0.5 and *θ_B_* ∈ (0.1, 0.9). The upper dashed line at 0.8 represents the desired power level, and the lower dashed line at 0.1 represents the nominal significance level.

Fig. 1 illustrates the statistical power, and type I error, of a study with 75 observations, where the result for *θ_A_* = *θ_B_* corresponds to the type I error rate. It can be seen that the fixed randomised design attains the highest power for all scenarios, as expected since this design aims to maximise the power and prioritise future patients.

In contrast, the power of the DP design, and the WI policy, is drastically reduced, even for large treatment differences. This is what we would expect since it is not possible to maximise both power and patient successes simultaneously, and unlike the fixed design, the optimal design aims to maximise the expected number of successes within the trial. Therefore, although the DP and WI designs are able to identify the superior treatment arm, they are unable to do so with sufficient statistical significance. We can see that the power of these designs lies below 0.3 for all *θ_B_* ∈ (0.1, 0.9), confirming that they are severely underpowered. As a result, they are clearly unsuitable to implement in practice.

Fig. 1 also shows that although the power of the RDP design is not as high as that of the fixed design, it greatly improves on the power of the other bandit designs aforementioned, and even exceeds the 0.8 level (illustrated by the upper dashed line) for some scenarios. Our proposed CRDP achieves even better power, with its power values lying much closer to those for the fixed design than the other bandit designs.

The obvious patterns, such as the power increasing with the size of the treatment difference for each design, are apparent in Fig. 1. Furthermore, additional evaluations for other sample sizes show similar patterns and can be seen in Fig. A.10 of the Appendix A.6.

Turning our attention to the type I error rates, we see that the type I error rate of both the DP and WI designs lies markedly below the nominal significance level at 0.1 (illustrated by the lower dashed line in Fig. 1) and is therefore greatly deflated for both designs. However, all of the other designs attain similar, higher observed type I error rates which are much closer to the nominal significance level and thus have better controlled type I error rates.

### 4.2 Patient benefit

**Fig. 2:**
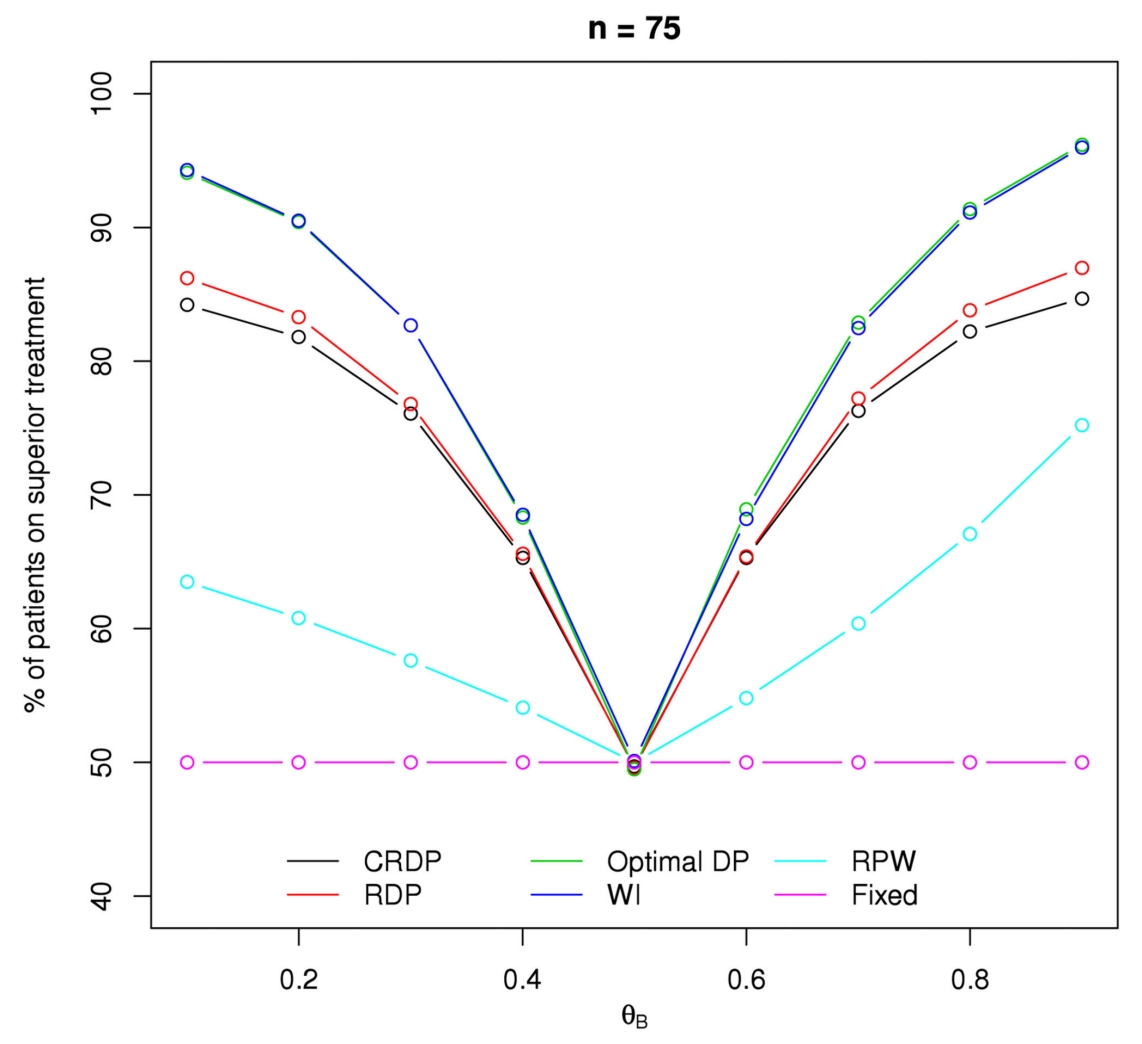
The percentage of patients on the superior treatment arm for each design when *n* = 75, *θ_A_* = 0.5 and *θ_B_* ∈ (0.1, 0.9).

Fig. 2 shows the percentage of patients (out of a total of 75) that receive the superior treatment within the trial. Note that when *θ_A_* = *θ_B_*, we define treatment *A* as the superior treatment for illustrative purposes and all designs show that approximately 50% of patients receive the superior treatment in this case, as expected.

The DP and WI designs perform the best, resulting in the highest percentage of patients receiving the superior treatment. This is not at all surprising considering they are designed to maximise the expected total reward (patient successes) within the trial in order to satisfy the patient benefit criterion.

At the other extreme, by design, the fixed randomised design allocates only 50% of the patients to the superior treatment in every scenario. Although the RPW does outperform the fixed design in terms of the patient benefit, the percentage of patients that are on the superior treatment is still much lower compared to all the other designs.

Fig. 2 shows that the RDP and CRDP designs perform very well and the percentage of patients receiving the superior treatment is still sufficiently high, with the CRDP line lying slightly below the RDP line due to the addition of the constraint. The largest difference between our proposed CRDP and DP designs is approximately 10%, which occurs at either end of the plot when the size of the treatment difference is at its largest. Moreover, our proposed CRDP design allocates a maximum of approximately 21% and 35% more patients to the superior treatment than the RPW and fixed designs, respectively.

For all designs (excluding the fixed), Fig. 2 shows that the percentage of patients allocated to the superior treatment increases with the magnitude of the treatment difference, with the higher values occurring at the tails of the graph which correspond to the larger treatment differences. Furthermore, similar patterns are observed for other sample sizes; see Fig. A.11 in Appendix A.6.

### 4.3 Average Bias

**Fig. 3:**
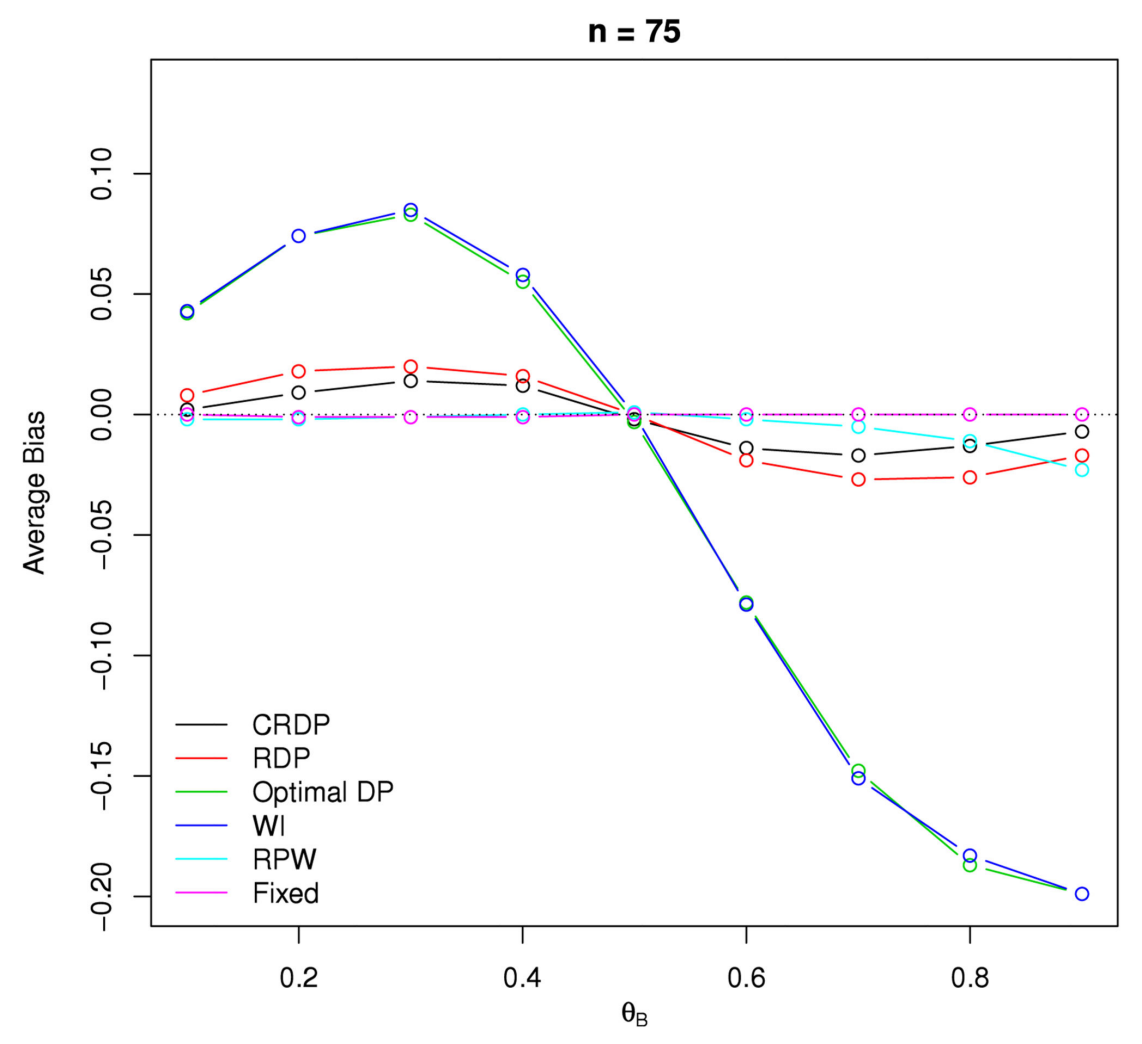
The average bias of the treatment effect estimator when *n* = 75, *θ_A_* = 0.5 and *θ_B_* ∈ (0.1, 0.9).

Fig. 3 shows the average bias of the sample proportion as an estimator for the treatment effect, as defined by (5), in a study with 75 observations. We see that the fixed randomised design produces the best result in terms of the bias, with its associated estimator attaining zero bias for all scenarios, as it should.

At the other extreme, the DP and WI designs exhibit the largest statistical bias with a maximum absolute value of 0.2 occurring when *θ_B_* = 0.9. Therefore, the corresponding estimates following such bandit designs will be biased due to the underlying dependence structure induced in the resulting observations. This is reflected in Table 1 which directly reports the raw estimates of the success probabilities, ${\hat{\theta }}_{A}$ and ${\hat{\theta }}_{B}.$ Table 1 shows that in the DP design, the estimate of the success probability for the inferior arm is substantially underestimated. The estimate for the superior arm is also underestimated, but less than for the inferior arm, particularly when the treatment difference is relatively small. This implies that the estimate of the treatment difference, $\hat{\Delta }$, is generally overestimated. Since bandit designs allocate fewer patients to the inferior treatment, it makes sense that the estimate corresponding to this arm is worse than that of the superior arm because there are fewer observations to base the inference on.

Once randomisation is incorporated into the DP design, we see that the bias is drastically reduced across all scenarios, with a maximum absolute value of 0.027 which is 85% smaller than the worst-case bias of the other bandit designs. Moreover, our proposed CRDP design performs significantly better than the RDP, reducing the bias of the treatment effect estimator even further. In fact, the bias values for our proposed CRDP are very close to zero for all scenarios with a maximum bias value of only 0.014 which is 93% smaller than the worst-case bias for the DP design. As such, the bias following our proposed CRDP is negligible compared to the very large bias exhibited by the other bandit designs and hence, the estimate of the treatment effect following our proposed CRDP design is essentially mean-unbiased. Again, this is reflected in Table 1 which shows that in our proposed CRDP design, ${\hat{\theta }}_{A}$ and ${\hat{\theta }}_{B}.$ are now much closer to their true values. Moreover, there is a large improvement in the estimate of the success probability for the inferior arm compared to the DP design since it is now only slightly underestimated.

Note that we can clearly see from Fig. 3 that all designs correctly attain a bias of zero for the treatment effect estimate when *θ_A_* = *θ_B_*. Similar results for different *n* are provided in Fig. A.12 of Appendix A.6.

### 4.4 Mean squared error

**Fig. 4:**
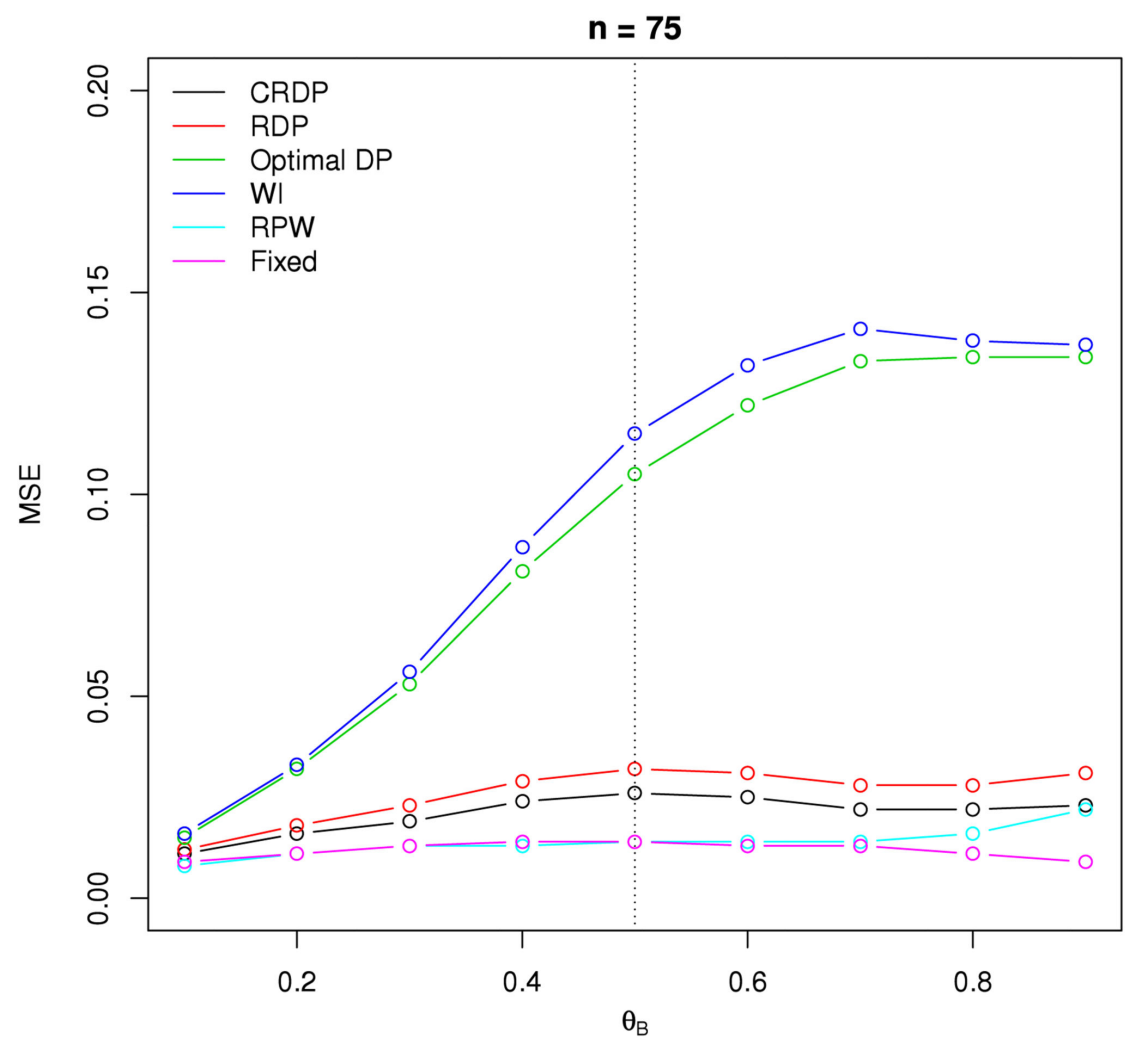
The mean squared error of the treatment effect estimator when *n* = 75, *θ_A_* = 0.5 and *θ_B_* ∈ (0.1, 0.9).

Fig. 4 shows the mean squared error (MSE) of the treatment effect estimator, as defined by (6), for a study with 75 observations. The fixed randomised design results in the smallest MSE, with values fairly constant and close to zero for all scenarios.

The DP and WI designs exhibit the largest MSE values, with the MSE of the WI design exceeding those of the DP design for all scenarios. This is a direct consequence of the large bias observed in Fig. 3. Moreover, these designs experience the largest increase in MSE as *θ_B_* increases from 0.1 to 0.7, after which point they remain fairly constant. Specifically, as *θ_B_* increases from 0.1 to 0.7, the MSE jumps from 0.016 to 0.141 for the WI design, and from 0.015 to 0.133 for the DP design. We also notice from Fig. 4 that the associated MSE plots for the DP and WI designs are not symmetric about *θ_B_* = 0.5 (represented by the dashed vertical line). This is a result of the variance of the estimator increasing markedly as *θ_B_* increases from 0.1 to 0.6, in addition to the bias for the DP and WI being much larger for larger values of *θ_B_*.

Once randomisation is incorporated into the DP, the MSE is reduced for all scenarios, from a worst-case value of 0.141 in the WI design to a worst-case value of 0.032 in the RDP design which is a 77.3% improvement. Moreover, our proposed CRDP design improves the MSE values even further, with a lower and an upper bound of 0.011 and 0.026, respectively. The majority of the MSE values lie around 0.030 for the RDP design and 0.020 for our proposed CRDP design. In contrast to the steep curves of the DP and WI designs, the MSE values associated with the RDP and our proposed CRDP designs remain fairly constant (as with the fixed and RPW designs), thus giving rise to the relatively flat curves visible in Fig. 4. Furthermore, we see that the curve corresponding to our proposed CRDP lies fairly close to the curve for the fixed design. Thus, the MSE values of the treatment effect estimator following our proposed CRDP design are comparable to that of the fixed design, staying close to zero for all scenarios, and are a huge improvement on those exhibited by the DP and WI bandit designs.

### 4.5 Overall performance

**Fig. 5:**
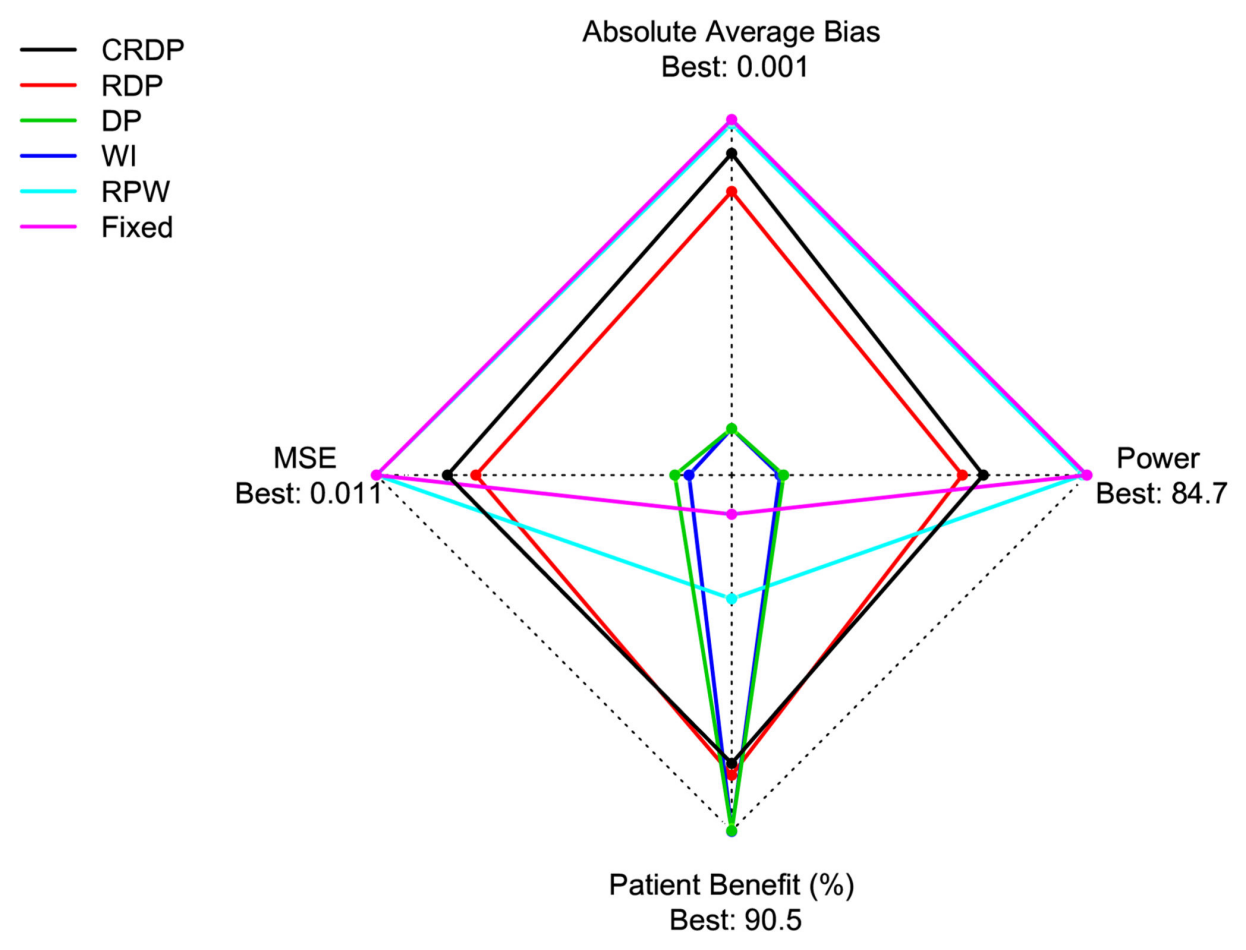
Star plot showing the performance of each design with respect to power, patient benefit, absolute average bias of the treatment effect estimator and MSE when *n* = 75, *θ_A_* = 0.5 and *θ_B_* = 0.2. The best achieved values for each performance measure are depicted at the outer edge. (Note that the average bias and MSE axes have been inverted so that the smaller (favourable) values are towards the outer edge, unlike the power and patient benefit axes which have their larger values towards the outer edge.)

Fig. 5 shows a star plot for each design against power, patient benefit, average bias of the treatment effect estimator and MSE in a trial with 75 patients when *θ_A_* = 0.5 and *θ_B_* = 0.2. The most desirable values lie towards the outer edge of the star plot with the least favourable values towards the centre. Fig. 5 summarises the key features of each design showing that the fixed design performs very well with respect to power, average bias and MSE but poorly with respect to patient benefit, whilst in contrast the DP design performs poorly with respect to power, average bias and MSE but very well with respect to patient benefit. Our proposed CRDP design, on the other hand, has values lying near to the outer edge of the star plot for power, average bias, MSE and patient benefit, thus showing that it performs well with respect to all of the performance measures. Table A.7 in Appendix A.5 reports additional combined measures that complement Fig. 5 to compare the designs.

### 4.6 CRDP patient allocation

**Fig. 6:**
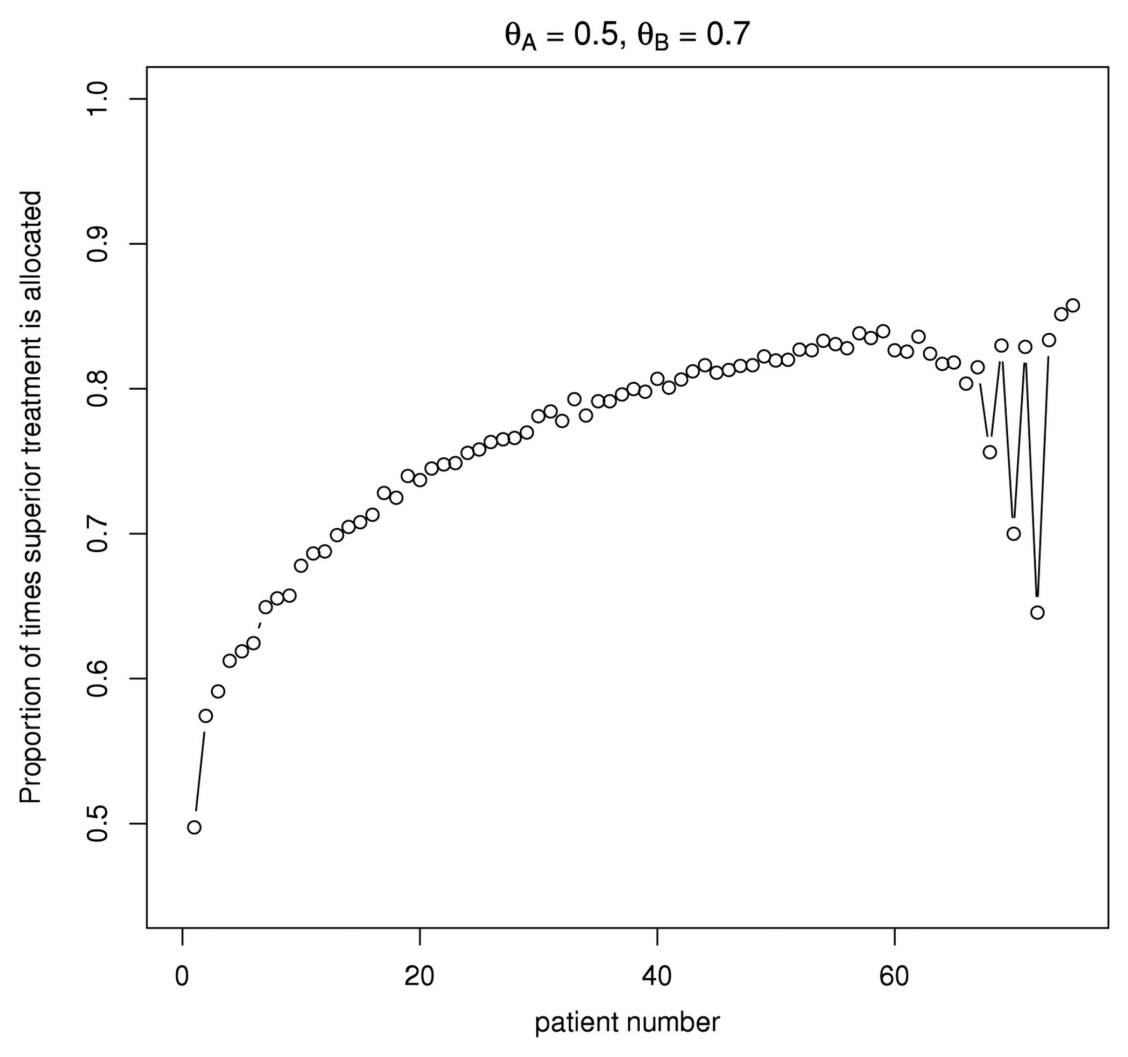
Probability of allocating a patient to treatment *B* for CRDP when *θ_A_* = 0.5 and *θ_B_* = 0.7 in a trial of size *n* = 75.

**Fig. 7:**
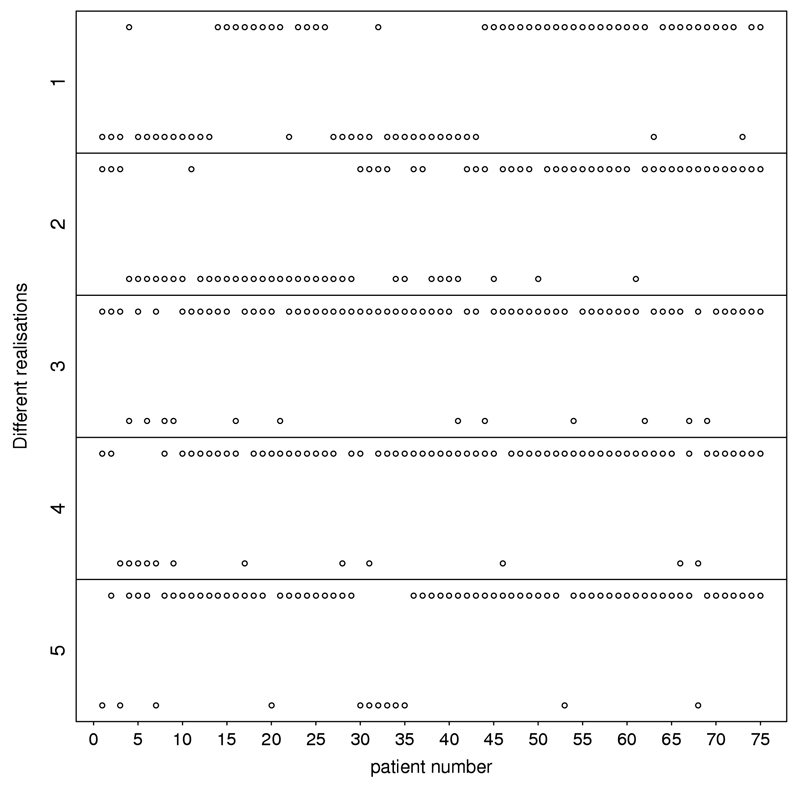
Patient allocations for CRDP when *θ_A_* = 0.5 and *θ_B_* = 0.7 in a trial of size *n* = 75 for five different trial realisations. Upper dots represent allocations to treatment *B* while lower dots represent allocations to treatment *A*.

Fig. 6 shows the average allocation probability to the superior treatment *B* under the CRDP design for every patient *t* in a trial with 75 patients when *θ_A_* = 0.5 and *θ_B_* = 0.7. This figure illustrates how the CRDP design adaptively allocates patients between the two treatments over time. The average allocation probability to a superior arm grows steadily through the trial towards the degree of randomisation selected (*p* = 0.9), but without reaching it in this scenario. As the trial approaches the treatment decisions for its final 15 patients, this probability markedly oscillates in order to satisfy the degree of constraining. This indicates that an important number of allocations to the inferior arm under the CRDP design tend to occur by the end of the trial rather than at the beginning of it. Fig. 7 also illustrates this point by plotting the observed patient allocations during five different trial realisations.

## 5 Discussion

In this paper, we evaluate different methods for allocating patients to treatments. The DP design performs very well when considering patient benefit compared to traditional fixed randomisation. However, this method suffers from an extremely low power to detect a significant treatment difference, biased estimates of the treatment effect and a large MSE. Moreover, it is completely deterministic and thus at risk of many possible sources of bias.

At the other extreme, fixed randomisation performs very well in terms of the statistical criteria, exhibiting high power, unbiased estimates of the treatment effect and small MSE. However, it allocates a large proportion of patients to the inferior treatment arm. This is particularly detrimental for rare, and fatal, diseases in which a substantial proportion of patients exhibiting the disease may be included in the trial and therefore the priority should be to treat these patients as effectively as possible.

We propose modifications to the DP design which overcome its current limitations and offer patient benefit advantages over a fixed randomised design by randomising in an optimal way and forcing a minimum number of patients on each arm. Our formal, mathematical approach grounded in decision theory creates a continuum of designs, with DP and fixed randomisation at the extremes, which offers freedom in choosing the most appropriate balance by fixing a degree of randomisation and a degree of constraining. This greatly increases the prospects of a bandit-based design being implemented in real clinical trial practice, particularly for trials involving rare diseases and small populations where the fixed randomisation approach is no longer the most appropriate design to use and is often not feasible due to the small sample sizes involved.

Our proposed CRDP design, with suggested degree of randomisation *p* = 0.9 and degree of constraining *𝓁* = 0.15*n*, seems to perform robustly in a range of simulated scenarios (not all of which are reported in the paper). The power is only slightly lower than with fixed randomisation, while almost as many patients are randomised to the superior treatment as in the DP design. Hence, this design strikes a very good balance in terms of the patient benefit and power trade-off, providing both power and ethical advantages, which acknowledges that clinical trials are multiple objective experiments.

The average bias and MSE of the treatment effect estimator following our proposed CRDP design are very low. It is well known that selection results in biased estimators (see e.g. Bauer et al., 2010). This is also true for group-sequential trials which are, however, routinely used in practice nowadays because the benefit from these designs can outweigh the bias incurred, particularly in the case of rare diseases. In order to make this assessment, it is important to determine the magnitude of the bias (as well as the benefits of the design) and hence the evaluations provided are essential for these novel methods to be applied in a real-life trial. In cases where the magnitude of the bias could be considered excessive, there exists a bias-corrected estimator that can be used (which comes at the price of a notably increased variability); see Bowden and Trippa (2015).

In this paper, we consider a two-armed trial with binary endpoints for simplicity, yet the principles used easily extend to multi-arm trials. An area of further work is to generalise the proposed design so it can be applied to other endpoints. In addition, a natural extension of this work is to modify the heuristic WI policy in a similar way as we have with the optimal DP design since index policies are conceptually more intuitive (we allocate the patient to the treatment with the highest index), and hence easier to communicate and be understood by clinicians. Moreover, the WI is potentially very important for the extension to more than two treatment arms since the DP quickly becomes computationally intractable while the WI is still feasible (Villar et al., 2015a).

In our proposed design, each patient’s response is used to inform the subsequent allocation decision. This relies on the assumption that patient responses become available before the next patient receives treatment (which would be the case if patient responses were quickly observed, for example). In many clinical trial settings, this is unrealistic because often a treatment takes a substantial length of time to induce a response and so it is very likely that the accrual rate will exceed the response rate. However, in a rare disease setting, the accrual rate is likely to be relatively slow with some patients being recruited over several years, and hence this assumption would be reasonable. Further research is required to address the problem of incorporating delayed responses into bandit-based designs which would increase the generalisability of our proposed design.

Moreover, our proposed design can only be applied to relatively small-scale trials since the underlying backwards induction algorithm suffers from the curse of dimensionality (Bellman, 1961) and currently attains its practical limit at *n* = 200. Again, this is not an issue for a rare disease setting in which the number of patients available for participation in the trial is limited, or clinical trials involving children, for example, in which recruitment is challenging (Hampson et al., 2014). In fact, many Phase II trials have no more than 200 patients, even in common diseases.

Additional extensions of this work include considering the effect of changing the prior distribution assigned to the unknown success probabilities. For example, a Beta prior with carefully chosen parameters could alternatively be used if the investigator wishes to reflect a greater amount of knowledge or a bias in favour of a particular treatment, without increasing the complexity of the problem. See Hampson et al. (2014) in which the unknown model parameters of the prior distribution are determined by eliciting expert opinion and incorporating historical data from a related trial.

## Supplementary Material

Appendix

## Figures and Tables

**Table 1:** The estimates of success probabilities, ${\hat{\theta }}_{A}$ and ${\hat{\theta }}_{B}$, and corresponding standard errors (s.e.) for the success probabilities of treatments *A* and *B*, respectively, compared to their true values *θ_A_* and *θ_B_*. These results correspond to the scenario in which *n* = 75, *θ_A_* = 0.5 and *θ_B_* ∈ (0.1, 0.9).

True		Fixed		DP		CRDP	
*θ_A_*	*θ_B_*	${\hat{\theta }}_{A}$ (s.e.)	${\hat{\theta }}_{B}$ (s.e.)	${\hat{\theta }}_{A}$ (s.e.)	${\hat{\theta }}_{B}$ (s.e.)	${\hat{\theta }}_{A}$ (s.e.)	${\hat{\theta }}_{B}$ (s.e.)
0.500	0.100	0.500 (0.083)	0.100 (0.050)	0.498 (0.062)	0.057 (0.096)	0.499 (0.064)	0.097 (0.085)
0.500	0.200	0.500 (0.083)	0.201 (0.065)	0.493 (0.080)	0.119 (0.132)	0.496 (0.070)	0.187 (0.105)
0.500	0.300	0.500 (0.083)	0.301 (0.075)	0.474 (0.118)	0.191 (0.156)	0.489 (0.084)	0.275 (0.109)
0.500	0.400	0.500 (0.083)	0.401 (0.080)	0.434 (0.162)	0.279 (0.176)	0.475 (0.098)	0.364 (0.107)
0.500	0.500	0.500 (0.083)	0.500 (0.082)	0.386 (0.192)	0.389 (0.192)	0.462 (0.105)	0.464 (0.106)
0.500	0.600	0.500 (0.083)	0.600 (0.080)	0.340 (0.216)	0.518 (0.193)	0.461 (0.111)	0.575 (0.099)
0.500	0.700	0.500 (0.083)	0.699 (0.075)	0.303 (0.240)	0.652 (0.172)	0.472 (0.123)	0.689 (0.080)
0.500	0.800	0.500 (0.083)	0.800 (0.065)	0.290 (0.266)	0.780 (0.129)	0.484 (0.136)	0.797 (0.058)
0.500	0.900	0.500 (0.083)	0.900 (0.049)	0.291 (0.290)	0.895 (0.074)	0.493 (0.147)	0.900 (0.039)
