# Supplementary material for: A Bayesian adaptive design for clinical trials in rare diseases
Source: Comput Stat Data Anal. Author manuscript; Available in PMC 2017 Sep 1. (PMC5473477; doi:10.1016/j.csda.2016.09.006)
Supplement: Appendix [file NIHMS71936-supplement-Appendix.pdf]

Appendix A

A.1. Backward induction algorithm

- If $t = n$, there is nothing to do because all n patients have already been treated and their outcomes observed. Thus, $\mathcal{F}_n(\widetilde{s}_{A,n}, \widetilde{f}_{A,n}, \widetilde{s}_{B,n}, \widetilde{f}_{B,n}) = 0 \; \forall \, \widetilde{s}_{A,n}, \; \widetilde{f}_{A,n}, \; \widetilde{s}_{B,n}, \; \widetilde{f}_{B,n}$.
- If $t = n-1$, there is only one patient left to treat and interest is in determining which treatment to allocate to this patient $\forall \, \widetilde{s}_{A,n-1}, \; \widetilde{f}_{A,n-1}, \; \widetilde{s}_{B,n-1}, \; \widetilde{f}_{B,n-1}$ that sum to $n - 1$. There are two possibilities:
  - If treatment A is allocated to the remaining patient, then we compute the expectation
  $$\mathcal{F}^A_{n-1}(\widetilde{s}_{A,n-1}, \widetilde{f}_{A,n-1}, \widetilde{s}_{B,n-1}, \widetilde{f}_{B,n-1}) = \frac{\widetilde{s}_{A,n-1}}{\widetilde{s}_{A,n-1} + \widetilde{f}_{A,n-1}} \cdot 1 + \frac{\widetilde{f}_{A,n-1}}{\widetilde{s}_{A,n-1} + \widetilde{f}_{A,n-1}} \cdot 0,$$
  where $\frac{\widetilde{s}_{A,n-1}}{\widetilde{s}_{A,n-1}+\widetilde{f}_{A,n-1}}$ is the expectation of θ_A with respect to a Beta$(\widetilde{s}_{A,n-1}, \widetilde{f}_{A,n-1})$ distribution, and $\frac{\widetilde{f}_{A,n-1}}{\widetilde{s}_{A,n-1}+\widetilde{f}_{A,n-1}}$ is the probability of a failure if treatment A is allocated.
  - Alternatively, if treatment B is allocated to the remaining patient, then we compute the expectation
  $$\mathcal{F}^B_{n-1}(\widetilde{s}_{A,n-1}, \widetilde{f}_{A,n-1}, \widetilde{s}_{B,n-1}, \widetilde{f}_{B,n-1}) = \frac{\widetilde{s}_{B,n-1}}{\widetilde{s}_{B,n-1} + \widetilde{f}_{B,n-1}} \cdot 1 + \frac{\widetilde{f}_{B,n-1}}{\widetilde{s}_{B,n-1} + \widetilde{f}_{B,n-1}} \cdot 0,$$
  where $\frac{\widetilde{s}_{B,n-1}}{\widetilde{s}_{B,n-1}+\widetilde{f}_{B,n-1}}$ is the expectation of θ_B with respect to a Beta$(\widetilde{s}_{B,n-1}, \widetilde{f}_{B,n-1})$ distribution, and $\frac{\widetilde{f}_{B,n-1}}{\widetilde{s}_{B,n-1}+\widetilde{f}_{B,n-1}}$ is the probability of a failure if treatment B is allocated.

  Interest is in choosing the optimal allocation such that
  $$\mathcal{F}_{n-1}(\widetilde{s}_{A,n-1}, \widetilde{f}_{A,n-1}, \widetilde{s}_{B,n-1}, \widetilde{f}_{B,n-1}) = \max\{\mathcal{F}^A_{n-1}(\widetilde{s}_{A,n-1}, \widetilde{f}_{A,n-1}, \widetilde{s}_{B,n-1}, \widetilde{f}_{B,n-1}), \; \mathcal{F}^B_{n-1}(\widetilde{s}_{A,n-1}, \widetilde{f}_{A,n-1}, \widetilde{s}_{B,n-1}, \widetilde{f}_{B,n-1})\}.$$
  Thus, if $\mathcal{F}^A_{n-1}(\widetilde{s}_{A,n-1}, \widetilde{f}_{A,n-1}, \widetilde{s}_{B,n-1}, \widetilde{f}_{B,n-1}) > \mathcal{F}^B_{n-1}(\widetilde{s}_{A,n-1}, \widetilde{f}_{A,n-1}, \widetilde{s}_{B,n-1}, \widetilde{f}_{B,n-1})$, then it is optimal to allocate the remaining patient to treatment A, and vice versa. If they are equal, then both treatments are optimal choices.
- The next step is if $t = n - 2$, i.e. when there are two remaining patients to be allocated. To determine which treatment to allocate to patient $n - 1$, there are two possibilities:
  - If treatment A is allocated to patient $n - 2$, then we compute the expectation
  $$\begin{aligned}\mathcal{F}^A_{n-2}(\widetilde{s}_{A,n-2}, \widetilde{f}_{A,n-2}, \widetilde{s}_{B,n-2}, \widetilde{f}_{B,n-2}) &= \frac{\widetilde{s}_{A,n-2}}{\widetilde{s}_{A,n-2} + \widetilde{f}_{A,n-2}} \cdot \left(1 + \mathcal{F}_{n-1}(\widetilde{s}_{A,n-2} + 1, \widetilde{f}_{A,n-2}, \widetilde{s}_{B,n-2}, \widetilde{f}_{B,n-2})\right) \\ &\quad + \frac{\widetilde{f}_{A,n-2}}{\widetilde{s}_{A,n-2} + \widetilde{f}_{A,n-2}} \cdot \left(0 + \mathcal{F}_{n-1}(\widetilde{s}_{A,n-2}, \widetilde{f}_{A,n-2} + 1, \widetilde{s}_{B,n-2}, \widetilde{f}_{B,n-2})\right).\end{aligned}$$
  - Similarly, if treatment B is allocated, then we compute the expectation
  $$\begin{aligned}\mathcal{F}^B_{n-2}(\widetilde{s}_{A,n-2}, \widetilde{f}_{A,n-2}, \widetilde{s}_{B,n-2}, \widetilde{f}_{B,n-2}) &= \frac{\widetilde{s}_{B,n-2}}{\widetilde{s}_{B,n-2} + \widetilde{f}_{B,n-2}} \cdot \left(1 + \mathcal{F}_{n-1}(\widetilde{s}_{A,n-2}, \widetilde{f}_{A,n-2}, \widetilde{s}_{B,n-2} + 1, \widetilde{f}_{B,n-2})\right) \\ &\quad + \frac{\widetilde{f}_{B,n-2}}{\widetilde{s}_{B,n-2} + \widetilde{f}_{B,n-2}} \cdot \left(0 + \mathcal{F}_{n-1}(\widetilde{s}_{A,n-2}, \widetilde{f}_{A,n-2}, \widetilde{s}_{B,n-2}, \widetilde{f}_{B,n-2} + 1)\right).\end{aligned}$$
- *et cetera.*

These steps are just iterations, and can be expressed more succinctly in the general form as follows.

If treatment A is allocated to the next patient, then the expected number of successes for patients $t + 1$ through n under an optimal policy is

$$\begin{aligned}\mathcal{F}^A_t(\widetilde{s}_{A,t}, \widetilde{f}_{A,t}, \widetilde{s}_{B,t}, \widetilde{f}_{B,t}) &= \frac{\widetilde{s}_{A,t}}{\widetilde{s}_{A,t} + \widetilde{f}_{A,t}} \cdot \left(1 + \mathcal{F}_{t+1}(\widetilde{s}_{A,t} + 1, \widetilde{f}_{A,t}, \widetilde{s}_{B,t}, \widetilde{f}_{B,t})\right) \\ &\quad + \frac{\widetilde{f}_{A,t}}{\widetilde{s}_{A,t} + \widetilde{f}_{A,t}} \cdot \mathcal{F}_{t+1}(\widetilde{s}_{A,t}, \widetilde{f}_{A,t} + 1, \widetilde{s}_{B,t}, \widetilde{f}_{Bt}).\end{aligned}$$

On the other hand, if treatment B is allocated to the next patient, then the expected total reward under an optimal policy is

$$\begin{aligned}\mathcal{F}^B_t(\widetilde{s}_{A,t}, \widetilde{f}_{A,t}, \widetilde{s}_{B,t}, \widetilde{f}_{B,t}) &= \frac{\widetilde{s}_{B,t}}{\widetilde{s}_{B,t} + \widetilde{f}_{B,t}} \cdot \left(1 + \mathcal{F}_{t+1}(\widetilde{s}_{A,t}, \widetilde{f}_{A,t}, \widetilde{s}_{B,t} + 1, \widetilde{f}_{B,t})\right) \\ &\quad + \frac{\widetilde{f}_{B,t}}{\widetilde{s}_{B,t} + \widetilde{f}_{B,t}} \cdot \mathcal{F}_{t+1}(\widetilde{s}_{A,t}, \widetilde{f}_{A,t}, \widetilde{s}_{B,t}, \widetilde{f}_{B,t} + 1).\end{aligned}$$

Therefore, $\mathcal{F}$ satisfies the recurrence

$$\mathcal{F}_t(\widetilde{s}_{A,t}, \widetilde{f}_{A,t}, \widetilde{s}_{B,t}, \widetilde{f}_{B,t}) = \max\left\{\mathcal{F}^A_t(\widetilde{s}_{A,t}, \widetilde{f}_{A,t}, \widetilde{s}_{B,t}, \widetilde{f}_{B,t}), \; \mathcal{F}^B_t(\widetilde{s}_{A,t}, \widetilde{f}_{A,t}, \widetilde{s}_{B,t}, \widetilde{f}_{B,t})\right\}.$$

Please cite this article in press as: Williamson, S.F., et al., A Bayesian adaptive design for clinical trials in rare diseases. Computational Statistics and Data Analysis (2016), http://dx.doi.org/10.1016/j.csda.2016.09.006

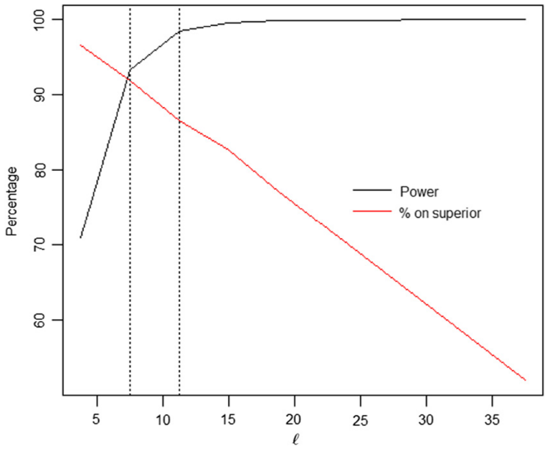

Fig. A.8. The effect of changing the degree of constraining, ℓ, on the power and percentage of patients on the superior treatment when $\theta_A = 0.2$ and $\theta_B = 0.8$ for the constrained DP design (without randomisation). The left and right dashed vertical lines correspond to $\ell = 0.10n$ and $\ell = 0.15n$ respectively, where $n = 75$ in this case.

Table A.2
Expected proportion of successes (EPS), run time in minutes (m) and seconds (s) and RAM memory requirements of the DP design (with uniform priors) on a standard laptop.

n	EPS	Run time	RAM
10	0.60218	0.01 s	0.1 MB
30	0.63066	1 s	6.2 MB
50	0.63993	6 s	47.7 MB
70	0.64485	24 s	183.2 MB
90	0.64799	1 m:04 s	0.56 GB
110	0.65020	2 m:22 s	1.1. GB
130	0.65186	4 m:37 s	2.1 GB
150	0.65316	8 m:03 s	3.86 GB
200	0.65547	25 m:20 s	11.9 GB

Table A.3
The effect of changing the degree of randomisation, p, on the performance measures when $n = 75$ and $\theta_A = \theta_B = 0.2$ for the RDP design (without the constraint).

p	Bias	MSE	Type I error	EPS	% on superior
0.5	0.000	0.004	0.035	0.200	50.0
0.6	−0.002	0.004	0.034	0.200	50.1
0.7	−0.001	0.005	0.027	0.200	50.2
0.8	0.000	0.005	0.022	0.200	50.0
0.9	0.000	0.006	0.008	0.200	50.2
1.0	0.001	0.008	0.000	0.200	49.7

Table A.2 illustrates the computational speed of the backwards induction algorithm to compute the allocation policy of the DP design on a standard laptop with 16 GB of RAM. The maximum trial size that can be computed on a standard laptop using R is 215. Although trials of sizes larger than 215 are very unlikely to occur in a rare disease context, computations of the DP design are feasible on a standard performance workstation (1 TB of RAM) for $215 < n < 600$. Trials of a size up to 3500 patients would be feasible with today's number #1 supercomputer (with 1.3 PB of RAM).

A.2. *Choosing the degree of constraining, ℓ*

Fig. A.8 illustrates the non-linearity of the power, based on which we recommend $\ell = 0.15n$ in our proposed CRDP design.

A.3. *Choosing the degree of randomisation, p*

Tables A.3, A.4, A.5, and A.6 illustrate the effect of randomisation, based on which we recommend $p = 0.9$ in our proposed CRDP design.

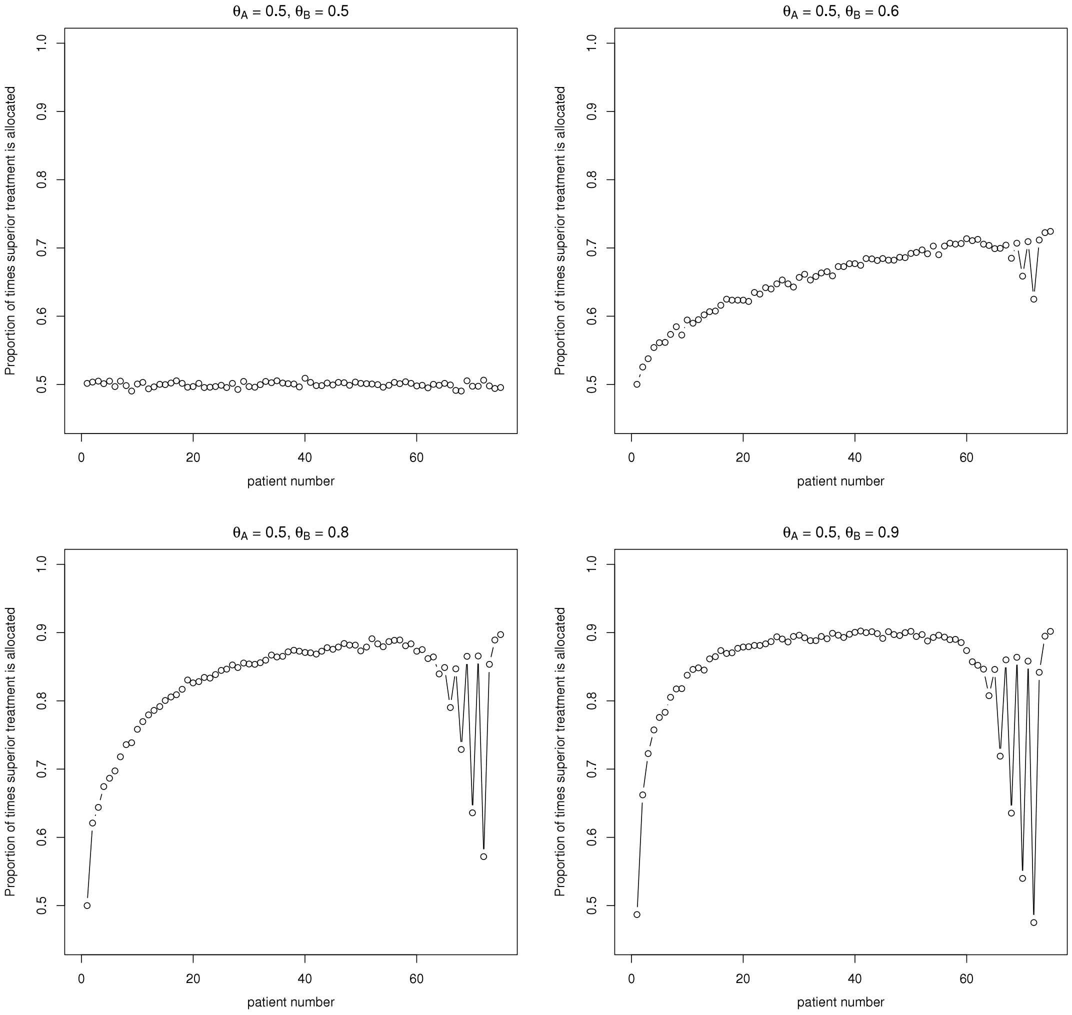

Fig. A.9. Probability of allocating a patient to treatment B for CRDP when $\theta_A = 0.5$ and $\theta_B = \{0.5, 0.6, 0.8, 0.9\}$ in a trial of size $n = 75$ estimated over 10,000 simulations.

Table A.4
The effect of changing the degree of randomisation, p, on the performance measures when $n = 75$, $\theta_A = 0.2$ and $\theta_B = 0.4$ for the RDP design (without the constraint).

p	Bias	MSE	Power	EPS	% on superior
0.5	−0.001	0.004	0.428	0.300	50.0
0.6	−0.002	0.005	0.406	0.315	57.3
0.7	−0.003	0.006	0.355	0.329	64.5
0.8	−0.007	0.007	0.289	0.344	71.4
0.9	−0.018	0.010	0.183	0.356	77.9
1.0	−0.058	0.017	0.021	0.368	83.6

Table A.5
The effect of changing the degree of randomisation, p, on the performance measures when $n = 75$, $\theta_A = 0.2$ and $\theta_B = 0.6$ for the RDP design (without the constraint).

p	Bias	MSE	Power	EPS	% on superior
0.5	−0.001	0.004	0.938	0.400	50.0
0.6	−0.002	0.005	0.935	0.437	59.1
0.7	−0.002	0.007	0.910	0.473	68.2
0.8	−0.005	0.009	0.830	0.509	77.3
0.9	−0.015	0.015	0.636	0.544	86.0
1.0	−0.089	0.03	0.070	0.577	94.2

Please cite this article in press as: Williamson, S.F., et al., A Bayesian adaptive design for clinical trials in rare diseases. Computational Statistics and Data Analysis (2016), http://dx.doi.org/10.1016/j.csda.2016.09.006

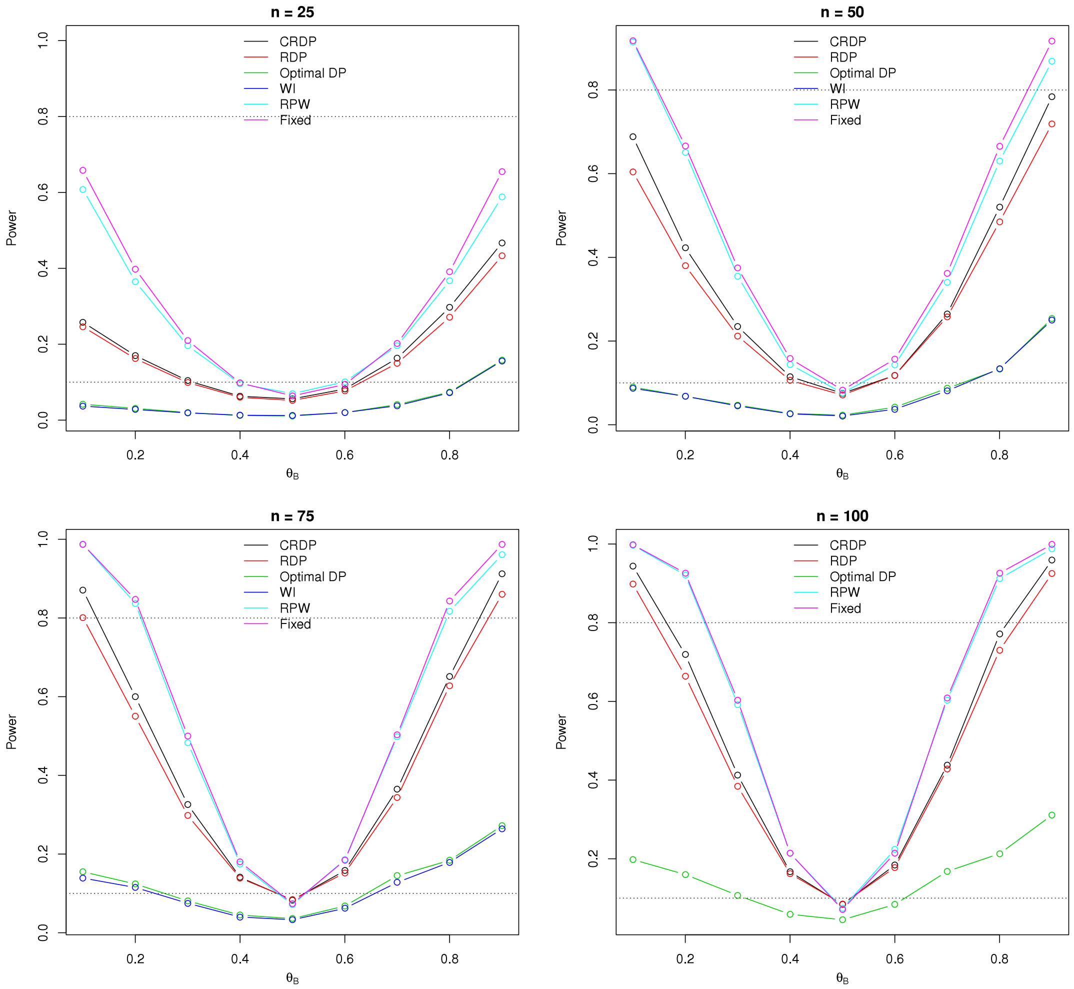

Fig. A.10. The changes in power and type I error for each design $\theta_A = 0.5$ and $\theta_B \in (0.1, \ 0.9)$ for varying sample sizes. The upper dashed line at 0.8 represents the desired power level, and the lower dashed line at 0.1 represents the nominal significance level. Note that WI is not available for $n = 100$ due to computational reasons.

Table A.6
The effect of changing the degree of randomisation, p, on the performance measures when $n = 75$, $\theta_A = 0.2$ and $\theta_B = 0.8$ for the RDP design (without the constraint).

p	Bias	MSE	Power	EPS	% on superior
0.5	−0.001	0.004	1.000	0.500	50.0
0.6	−0.001	0.005	1.000	0.557	59.6
0.7	−0.001	0.007	0.999	0.615	69.2
0.8	−0.004	0.010	0.995	0.672	78.8
0.9	−0.009	0.019	0.937	0.730	88.3
1.0	−0.100	0.043	0.118	0.786	97.6

A.4. *CRDP patient allocation: other scenarios*

Fig. A.9 complements Fig. 6 to show average allocation probabilities of our proposed CRDP design in other scenarios.

A.5. *Combined performance measures*

Table A.7 summarises the performance of the four key features (power, average bias, MSE and patient benefit) per design by showing the following measures: (i) sum of the distance of each key feature from the best achievable value (SDis), (ii) the

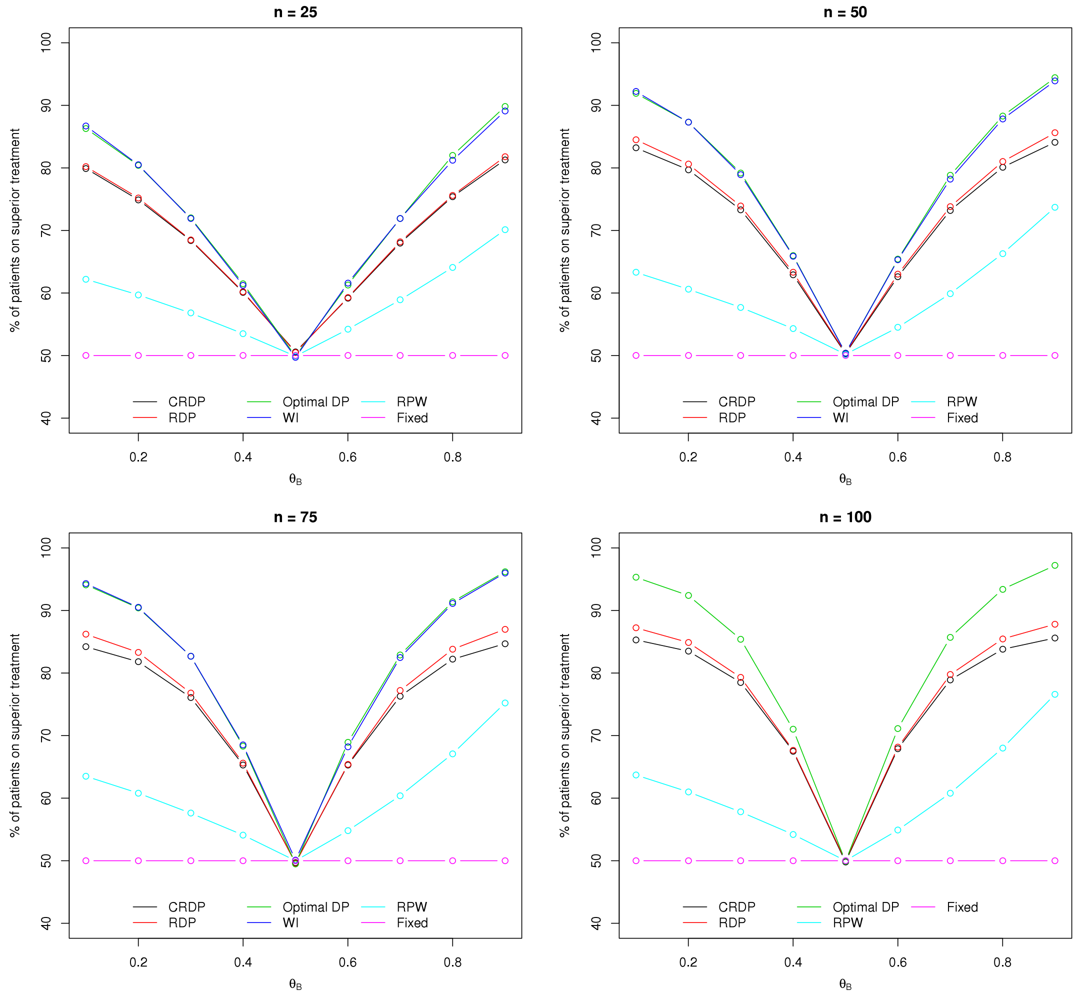

Fig. A.11. The percentage of patients on the superior treatment arm for each design when $\theta_A = 0.5$ and $\theta_B \in (0.1, \ 0.9)$ for varying sample sizes. Note that WI is not available for $n = 100$ due to computational reasons.

Table A.7
The summary measures of performance in terms of the four key features. SDis: sum of the distance of each key feature from the best achievable value; MD: maximum difference among each of the key features from the best achievable value; SDev: sum of the deviations of each key feature from the fixed randomisation design.

Design	SDis	MD	SDev
CRDP	32.925	24.7	53.513
RDP	36.936	29.7	63.009
DP	74.439	72.3	95.494
WI	73.307	73.2	113.695
RPW	30.714	29.7	11.801
Fixed	40.512	50.0	0

maximum difference among each of the four key features from the best achievable value (MD), (iii) sum of the deviations of each key feature from the fixed randomisation design (SDev).

A.6. Results for other sample sizes

Figs. A.10–A.12 complement Figs. 1–3, respectively, to compare the performance of our proposed CRDP design with alternative designs for different sample sizes.

Please cite this article in press as: Williamson, S.F., et al., A Bayesian adaptive design for clinical trials in rare diseases. Computational Statistics and Data Analysis (2016), http://dx.doi.org/10.1016/j.csda.2016.09.006

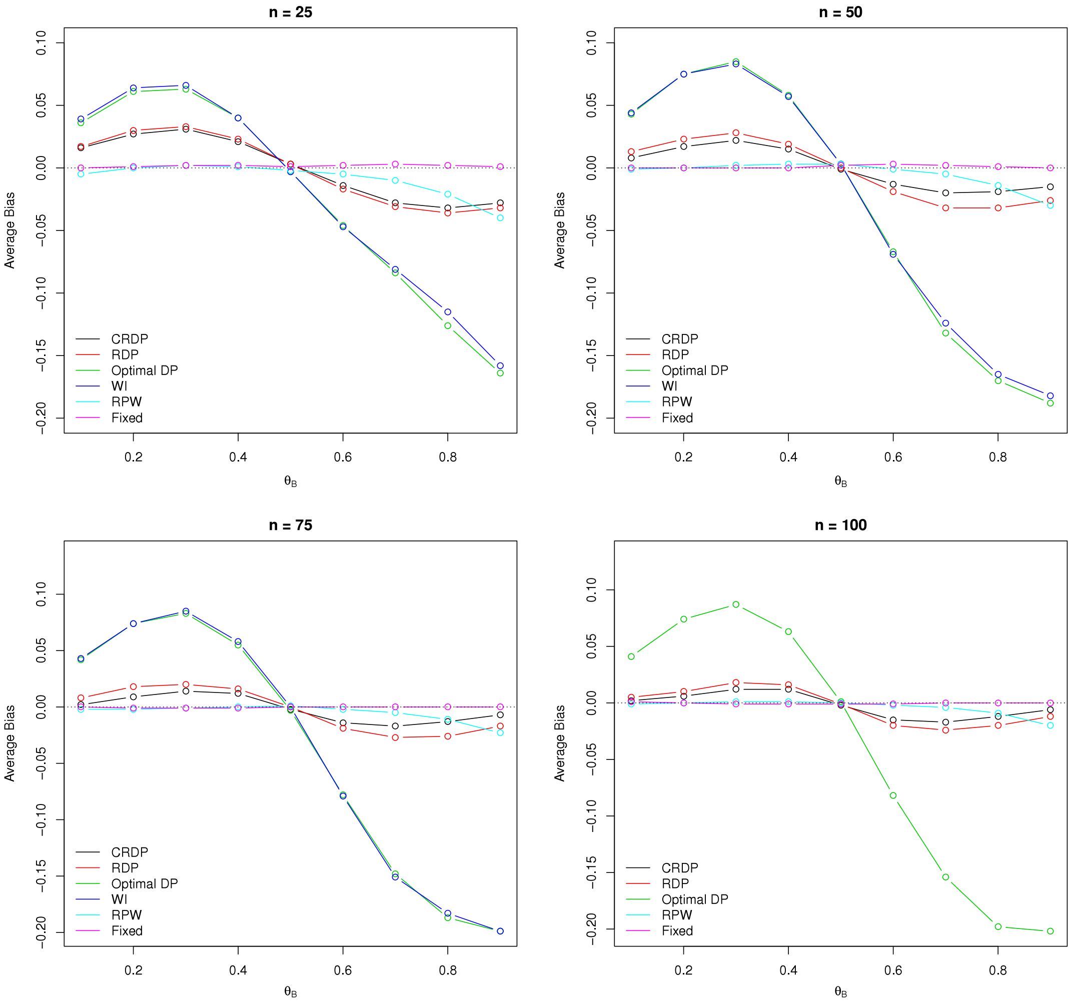

Fig. A.12. The average bias of the treatment effect estimator when $\theta_A = 0.5$ and $\theta_B \in (0.1, \ 0.9)$ for varying sample sizes. Note that WI is not available for $n = 100$ due to computational reasons.
